# Supplementary figures and images for: An eQTL-based approach reveals candidate regulators of LINE-1 RNA levels in lymphoblastoid cells
Source: PLoS Genet. 2024 Jun 7;20(6):e1011311. doi: 10.1371/journal.pgen.1011311 (PMC11189215; doi:10.1371/journal.pgen.1011311)

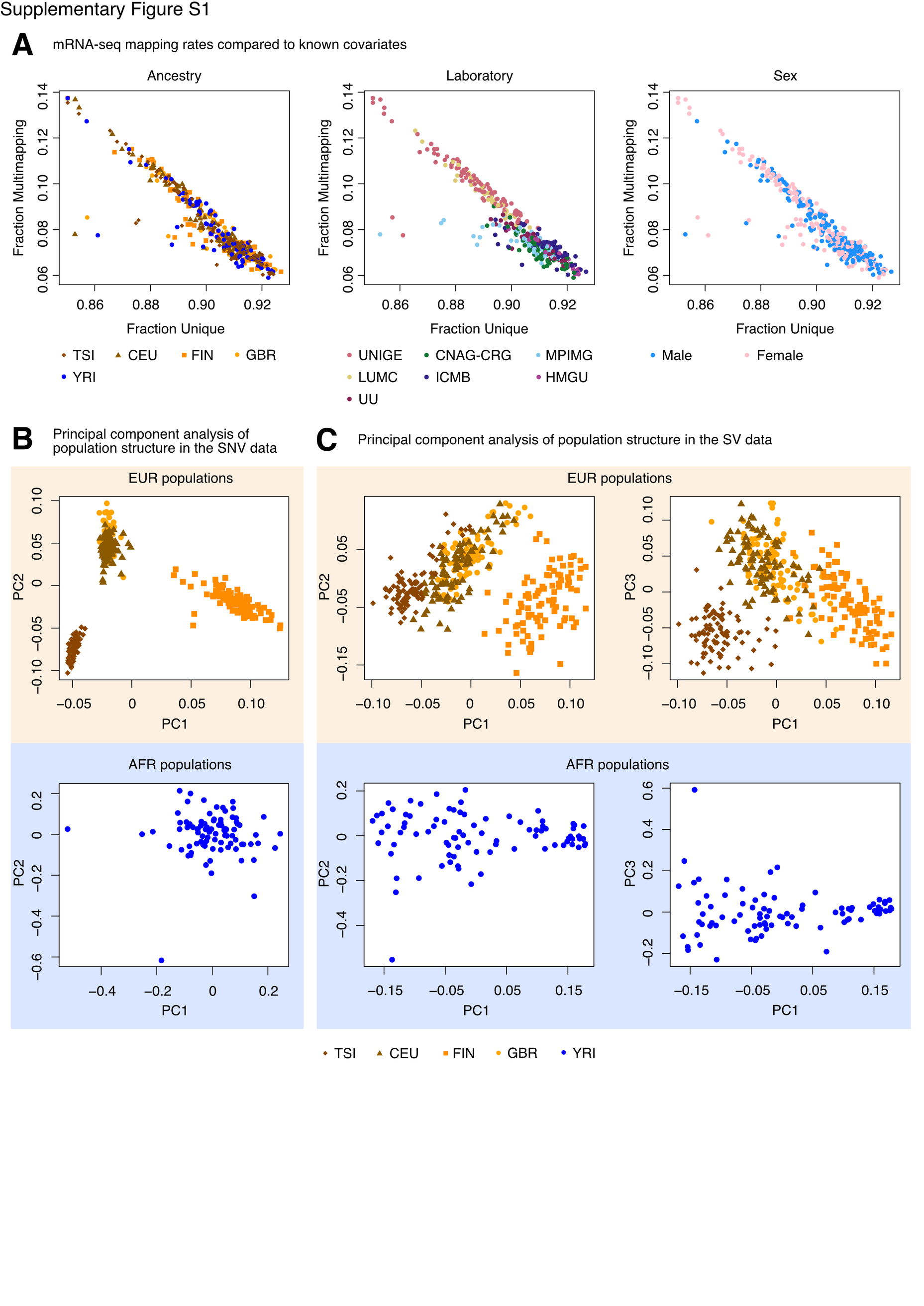

Supplement: S1 Fig — (A) Unique and multimapping fractions for the mRNA-sequencing data, color-coded by ancestry group, laboratory, or biological sex. For visual clarity, the plots were limited to samples with a minimum of 84% uniquely mapped reads, a limit which still captured the majority of samples analyzed. For ancestry, the following groups were analyzed: Tuscan (TSI), Northern Europeans from Utah (CEU), Finnish (FIN), British (GBR), and Yoruba (YRI). (B) PCA plots for pruned SNV genotype data from European or African samples, color-coded and shaped according to ancestry group. (C) PCA plots for pruned SV genotype data from European or African samples, color-coded and shaped according to ancestry group. (TIF) [file pgen.1011311.s001.tif]

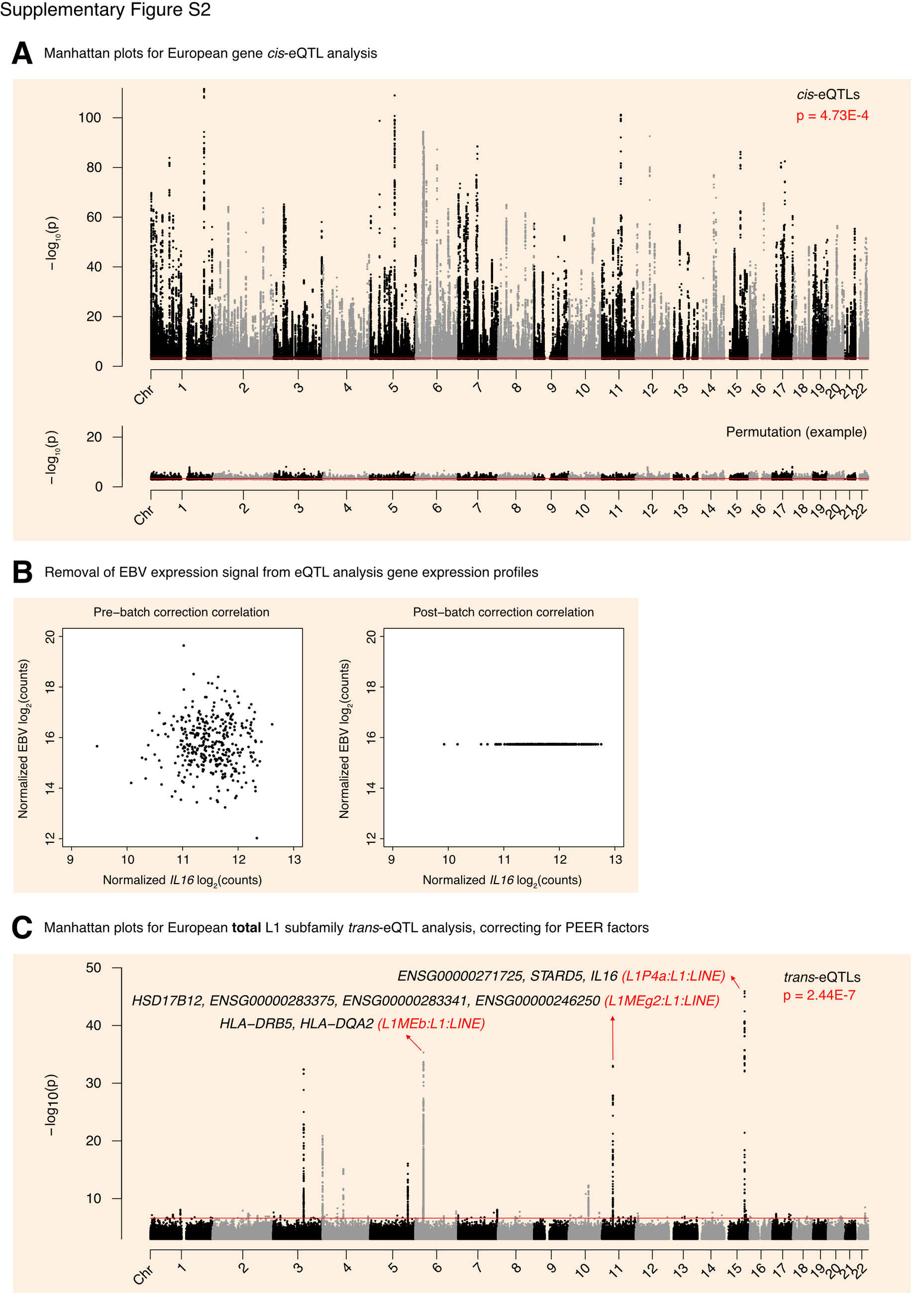

Supplement: S2 Fig — (A) A Manhattan plot for the gene cis-eQTL analysis in the European cohort. The dashed line at p = 4.75E-4 corresponds to an average empirical FDR < 0.05, based on 20 random permutations. One such permutation is illustrated in the bottom panel. The solid line at p = 4.73E-4 corresponds to a Benjamini-Hochberg FDR < 0.05. The stricter of the two thresholds, p = 4.73E-4, was used to define significant cis-eQTLs. (B) EBV expression as a function of IL16 expression prior to (left) and after (right) correcting for EBV expression. This correction was applied prior to the eQTL scan in order to avoid confounding SNV associations with differences in EBV expression. (C) A Manhattan plot for the L1 subfamily trans-eQTL analysis in the European cohort, after correcting for known covariates as well as 10 PEER factors. The genes and L1 subfamilies identified in the original analysis without PEER are listed next to the equivalent peaks in this analysis. The solid line at p = 2.44E-7 corresponds to a Benjamini-Hochberg FDR < 0.05, which was used to define significant trans-eQTLs. FDR: False Discovery Rate. (TIF) [file pgen.1011311.s002.tif]

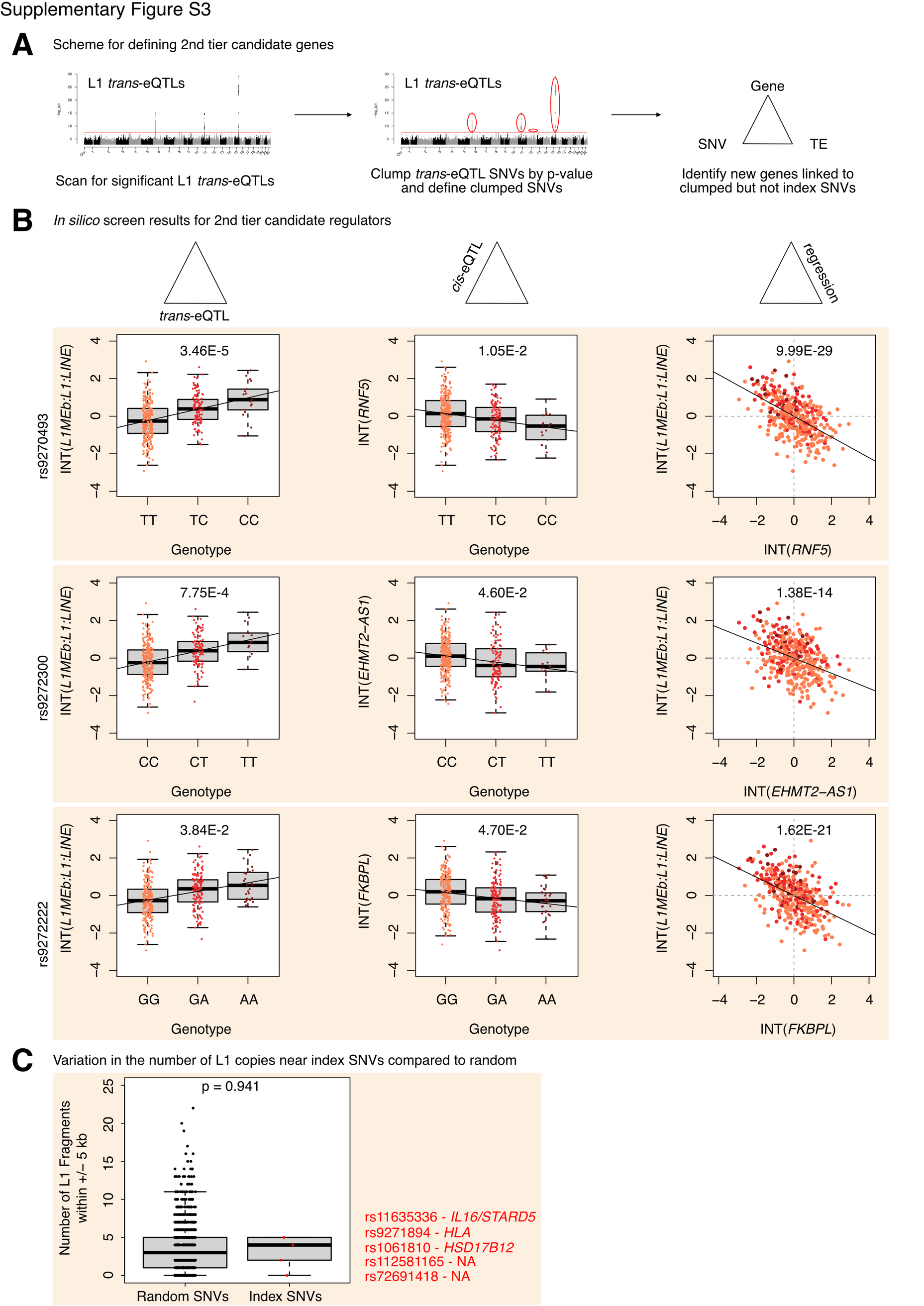

Supplement: S3 Fig — (A) A schematic for how 2nd tier candidate genes were defined. In short, these were genes in trios with clumped SNVs but not index SNVs at the top of each peak. (B) The three-part integration results for three genes—RNF5, EHMT2-AS1, FKBPL—that we considered second tier candidates for functional, in vitro testing. In the left column are the trans-eQTLs, in the middle column are the cis-eQTLs, and in the right column are the linear regressions for gene expression against L1 subfamily RNA levels. Expression values following an inverse normal transform (INT) are shown. The FDR for each analysis is listed at the top of each plot. (C) The number of L1 fragments near 1000 random SNVs or the 5 trans-eQTL index SNVs were calculated. A two-sample Wilcoxon test was run to determine whether there were significant differences between the two groups. FDR: False Discovery Rate. (TIF) [file pgen.1011311.s003.tif]

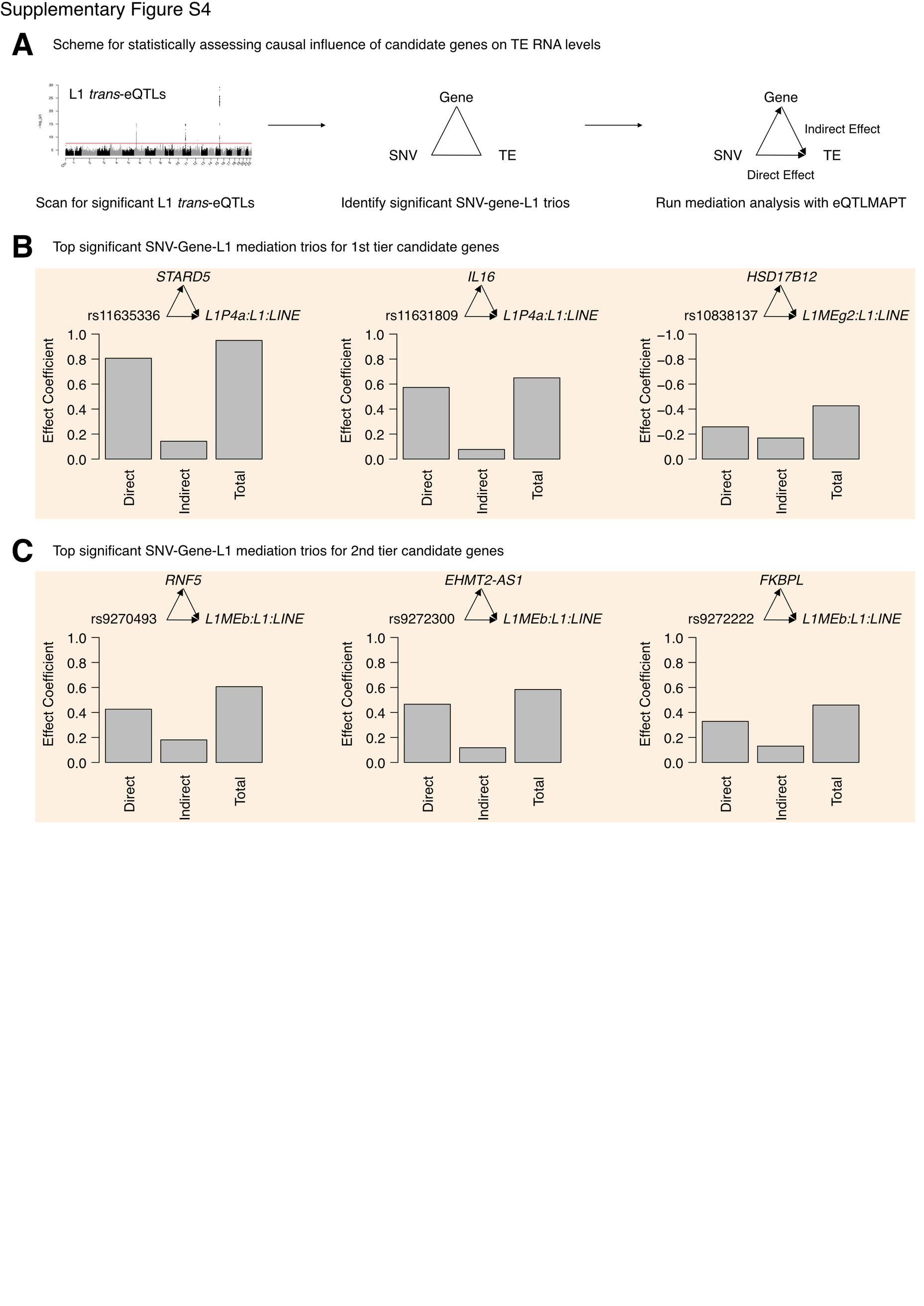

Supplement: S4 Fig — (A) Scheme for the mediation analysis. Mediation analysis tests the mechanistic model where a given gene, in cis to a given SNV, partially or fully mediates the effect that SNV has on TE RNA levels in trans. The direct, indirect, and total effects for (B) 1st tier candidate gene trios and (C) 2nd tier candidate gene trios are shown. Mediation was considered significant if the FDR-adjusted empirical p-value, calculated from 30,000 permutations, was < 5%. FDR: False Discovery Rate. (TIF) [file pgen.1011311.s004.tif]

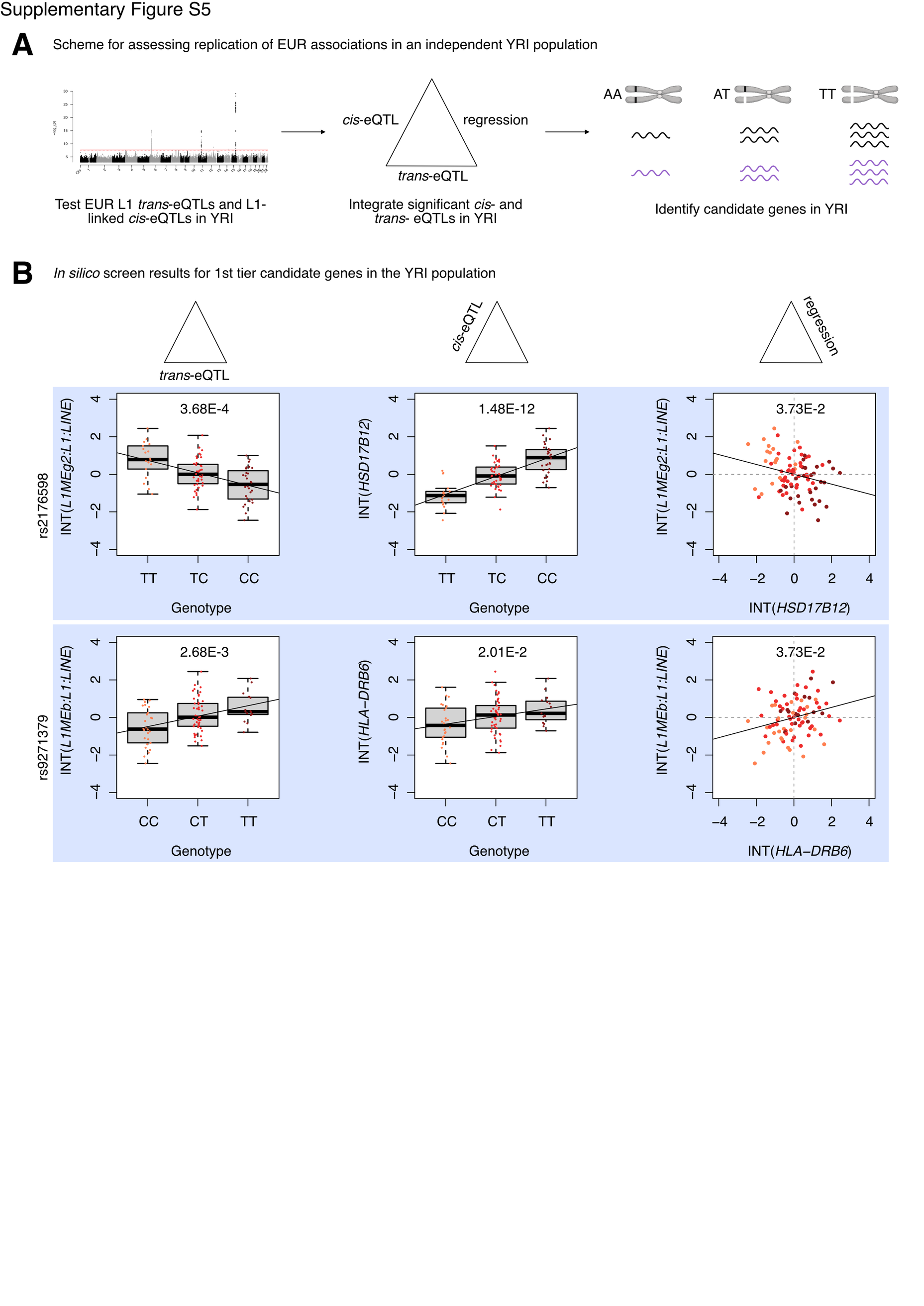

Supplement: S5 Fig — (A) A scheme for the in silico analysis carried out with an African cohort, which was similar to the analysis with the European cohort. Since the sample size was smaller than the European cohort, a targeted eQTL approach was undertaken, where we 1) only checked for replication of significant L1 trans-eQTLs and 2) only tested significant trans-eQTL SNVs for cis-association with genes that were L1-linked in the European cohort analysis. (B) The three-part integration results for two 1st tier candidate regulators in the African cohort—HSD17B12 and HLA-DRB6. In the left column are the trans-eQTLs, in the middle column are the cis-eQTLs, and in the right column are the linear regressions for gene expression against L1 subfamily expression. Expression values following an inverse normal transform (INT) are shown. The FDR for each analysis is listed at the top of each plot. FDR: False Discovery Rate. Panel (A) was created with BioRender.com. (TIF) [file pgen.1011311.s005.tif]

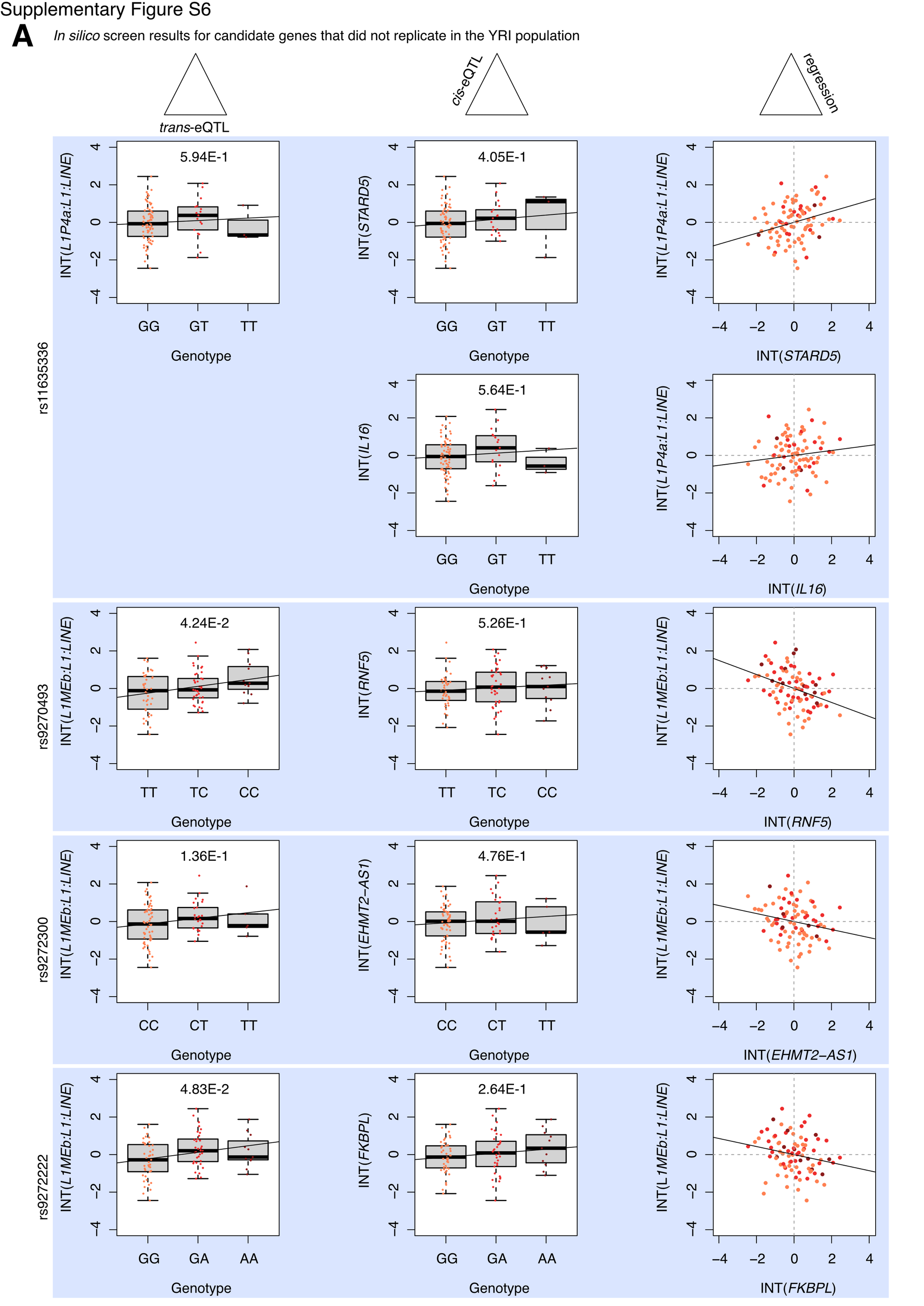

Supplement: S6 Fig — Many candidate genes from the in silico screen in the European cohort did not replicate in the African cohort, likely due to the much smaller sample size and relative rarity of homozygotes carrying 2 alternate alleles. (A) The three-part integration results for 1st and 2nd tier candidate regulators identified in the European cohort analysis and tested in the African cohort. In the left column are the trans-eQTLs, in the middle column are the cis-eQTLs, and in the right column are the linear regressions for gene expression against L1 subfamily expression. Expression values following an inverse normal transform (INT) are shown. The FDR is listed for each integration step except for the linear regressions since trios were filtered out before the regression step. FDR: False Discovery Rate. (TIF) [file pgen.1011311.s006.tif]

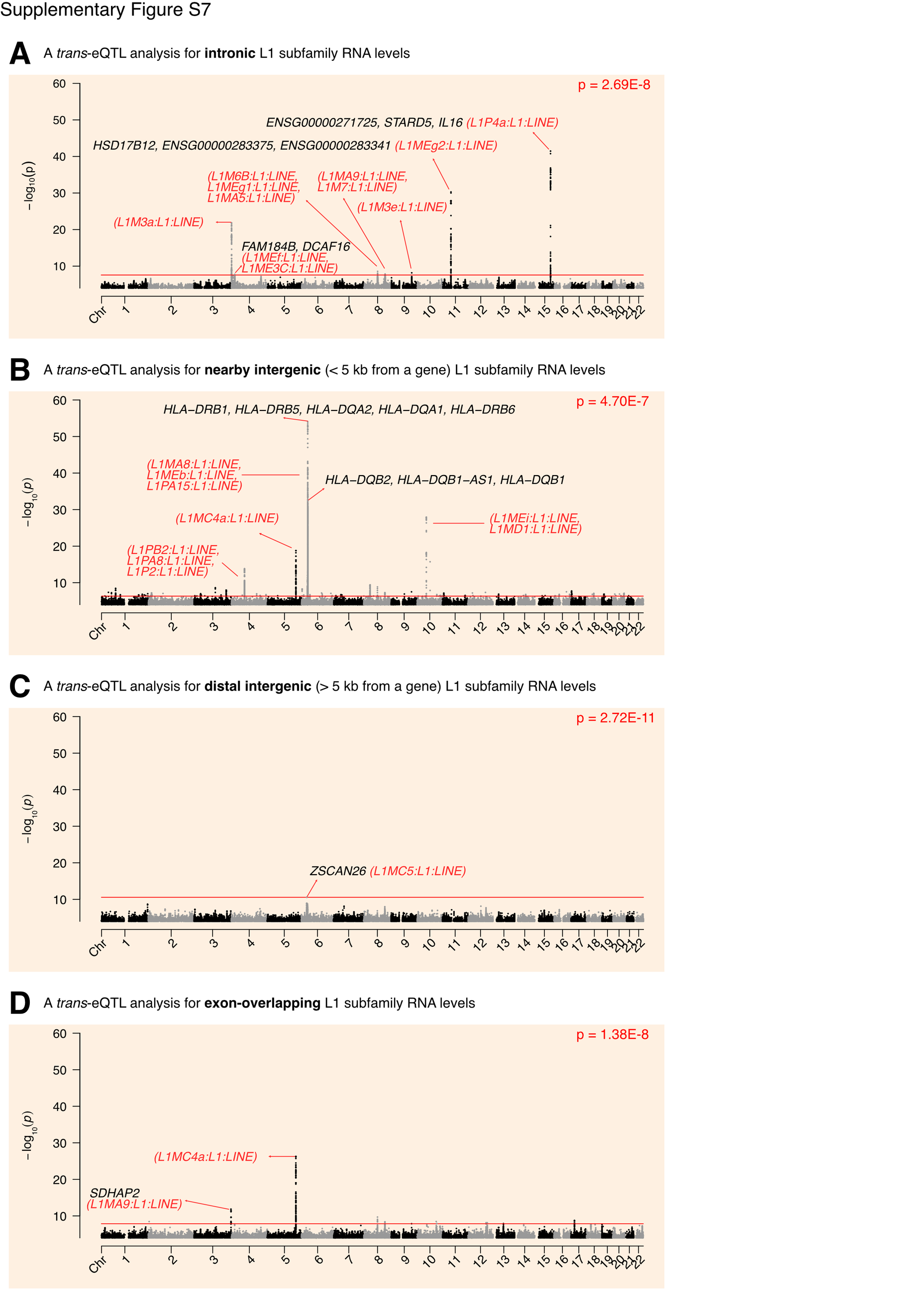

Supplement: S7 Fig — Transposon locus-specific quantifications were obtained using the TElocal package, and these were stratified by genomic region (i.e. intronic, nearby intergenic for loci < 5 kb from a gene, distal intergenic for loci > 5 kb from a gene, and exon-overlapping). Counts were then aggregated at the subfamily level, and the L1 eQTL scan was re-run using each of the four L1 expression profiles using the European cohort. The Manhattan plots for the (A) intronic L1 subfamily, (B) nearby intergenic L1 subfamily, (C) distal intergenic L1 subfamily, and (D) exon-overlapping L1 subfamily trans-eQTL analyses. For readability, only a subset of associated genes and L1s are highlighted in each plot. For regions with trans-eQTLs for multiple L1 subfamilies, we used an un-pointed line to depict the association between the listed L1 subfamilies and at least one SNV in that region. The solid red line in each plot corresponds to a Benjamini-Hochberg FDR < 0.05. FDR: False Discovery Rate. (TIF) [file pgen.1011311.s007.tif]

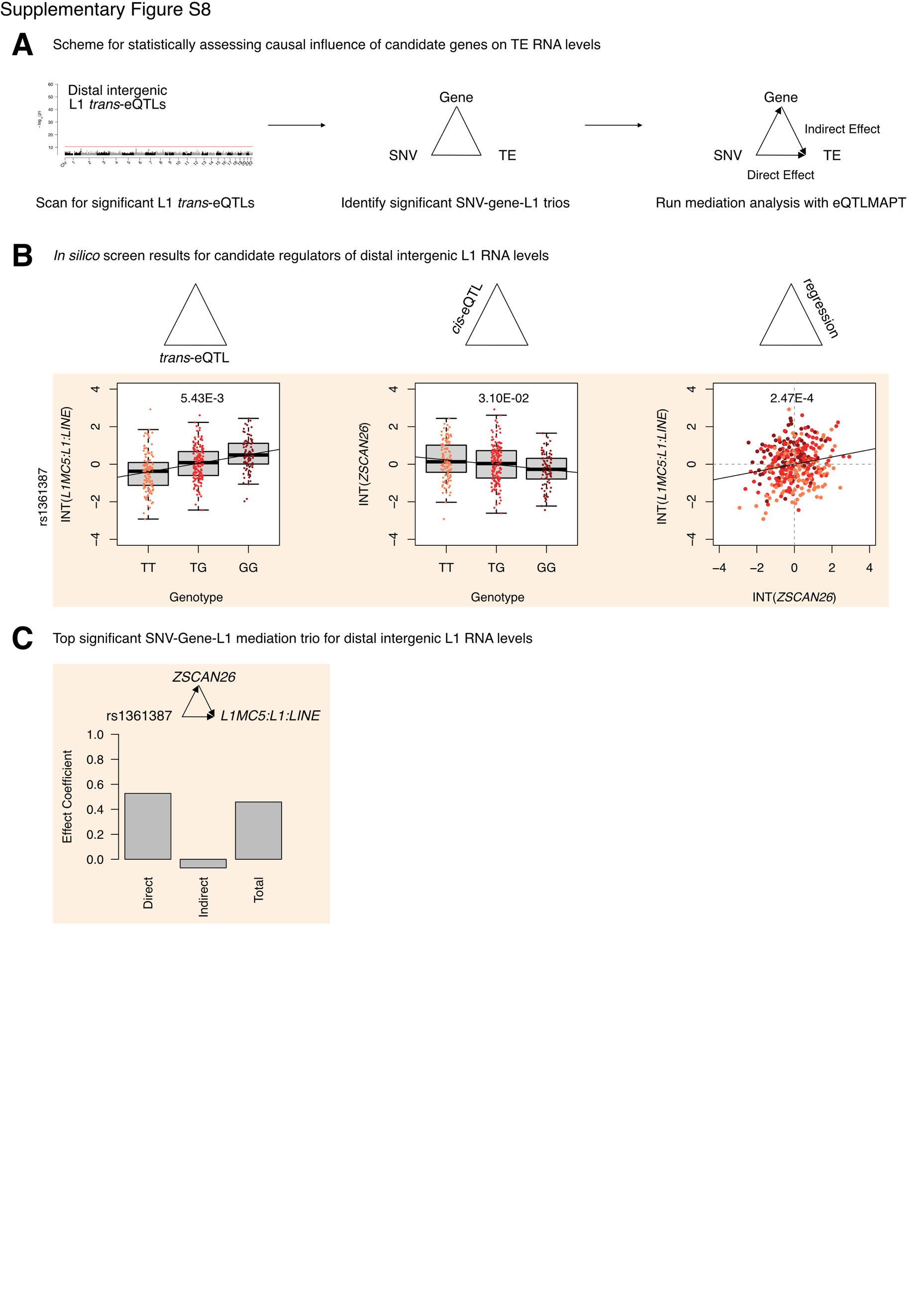

Supplement: S8 Fig — (A) Scheme for the mediation analysis. Mediation analysis tests the mechanistic model where a given gene, in cis to a given SNV, partially or fully mediates the effect that SNV has on TE expression in trans. (B) The three-part integration results for one protein-coding gene—ZSCAN26—that we considered a candidate regulator of distal intergenic L1 RNA levels. In the left column are the trans-eQTLs, in the middle column are the cis-eQTLs, and in the right column are the linear regressions for gene expression against L1 subfamily RNA levels. Expression values following an inverse normal transform (INT) are shown. The FDR for each analysis is listed at the top of each plot. (C) The ZSCAN26 SNV-gene-TE mediation trio results. Mediation was considered significant if the FDR-adjusted empirical p-value, calculated from 30,000 permutations, was < 5%. FDR: False Discovery Rate. (TIF) [file pgen.1011311.s008.tif]

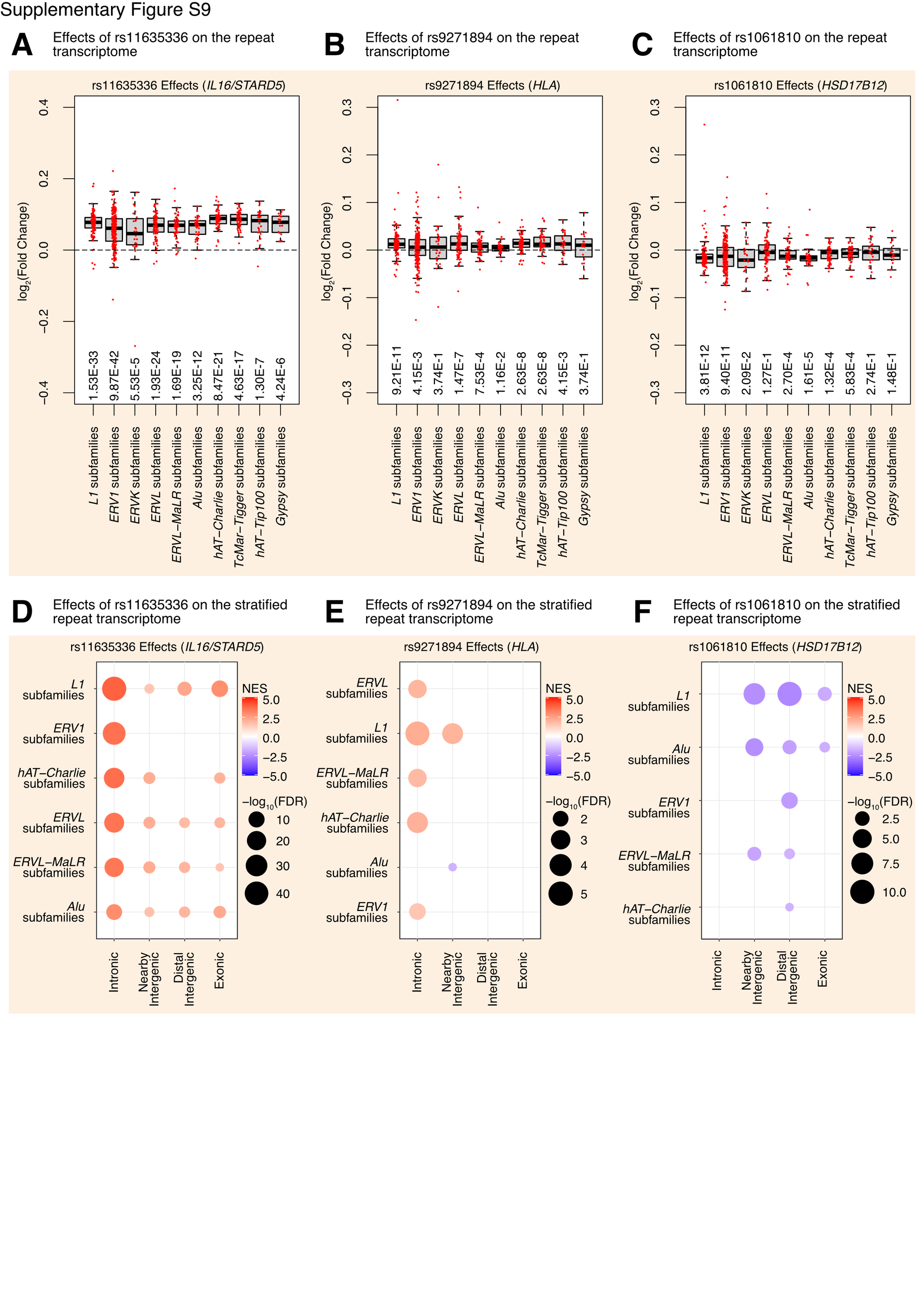

Supplement: S9 Fig — Box and whisker plots for the log2 fold changes of TE subfamilies (red dots), grouped by TE family, across genotypes for (A) rs11635336 (IL16/STARD5), (B) rs9271894 (HLA), and (C) rs1061810 (HSD17B12). A one-sample Wilcoxon test was run to determine whether changes were significantly different from 0. The FDR values from this test are listed at the bottom. GSEA analysis for top, differentially regulated TE family gene sets in different genomic regions (intronic, intergenic, exon-overlapping) across genotypes. The results for (D) rs11635336 (IL16/STARD5), (E) rs9271894 (HLA), and (F) rs1061810 (HSD17B12) are shown. In each bubble plot, the size of the dot represents the -log10(FDR) and the color reflects the normalized enrichment score. FDR: False Discovery Rate. (TIF) [file pgen.1011311.s009.tif]

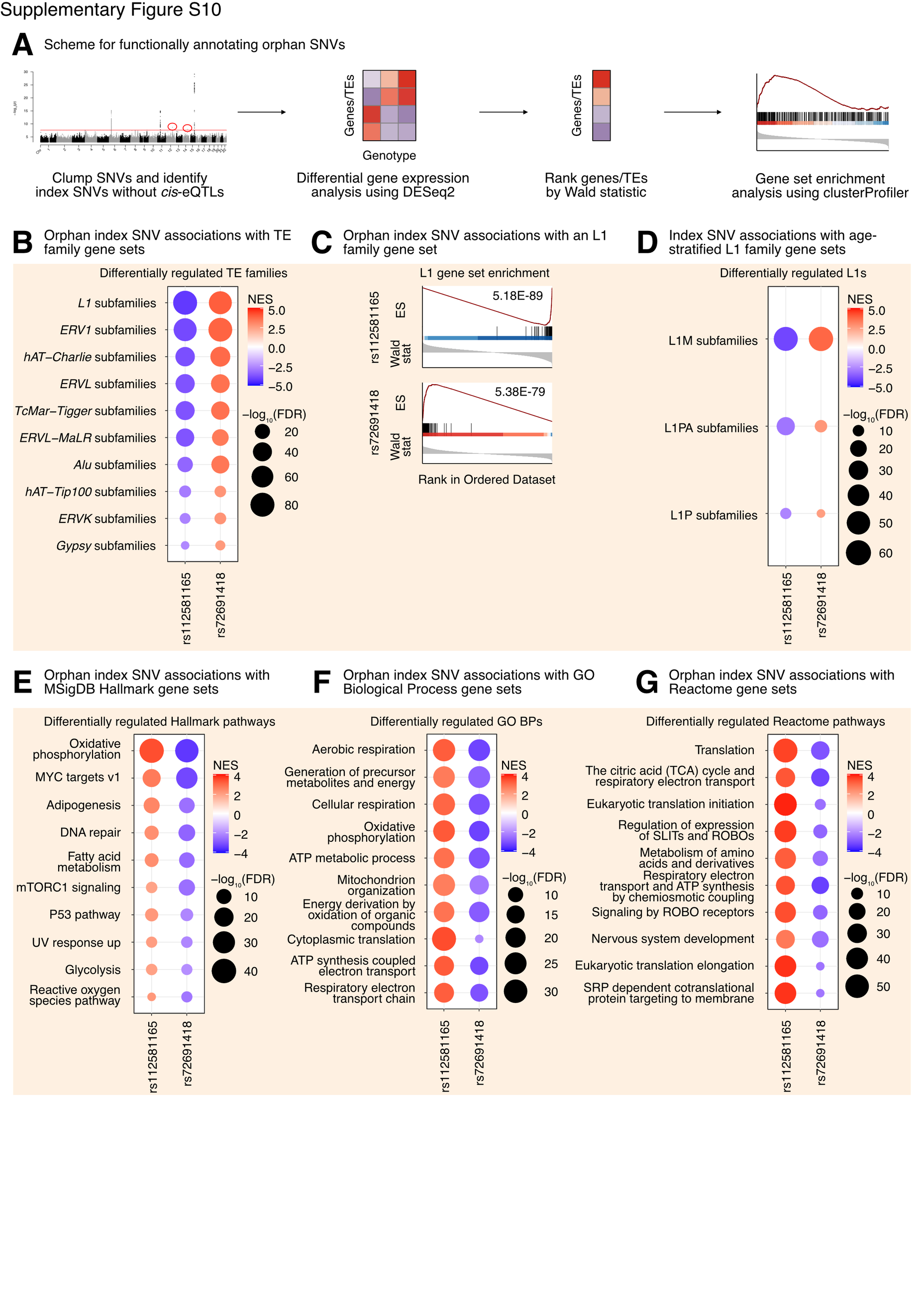

Supplement: S10 Fig — (A) Scheme for functionally annotating orphan index SNVs by GSEA. (B) GSEA analysis for shared, significantly regulated TE family gene sets across genotypes for rs112581165 and rs72691418. (C) GSEA plots for the L1 family gene set results summarized in (B). For these plots, the FDR value is listed. (D) GSEA analysis for shared, significantly regulated, evolutionary-age-stratified L1 gene sets across genotypes for rs112581165 and rs72691418. L1M subfamilies are the oldest, L1P subfamilies are intermediate, and L1PA subfamilies are the youngest. GSEA analysis for top, shared, concomitantly regulated (E) MSigDB Hallmark pathway, (F) GO Biological Process, and (G) Reactome pathway gene sets across genotypes for rs112581165 and rs72691418. Shared gene sets were ranked by combining p-values from each individual SNV analysis using Fisher’s method. In each bubble plot, the size of the dot represents the -log10(FDR) and the color reflects the normalized enrichment score. FDR: False Discovery Rate. (TIF) [file pgen.1011311.s010.tif]

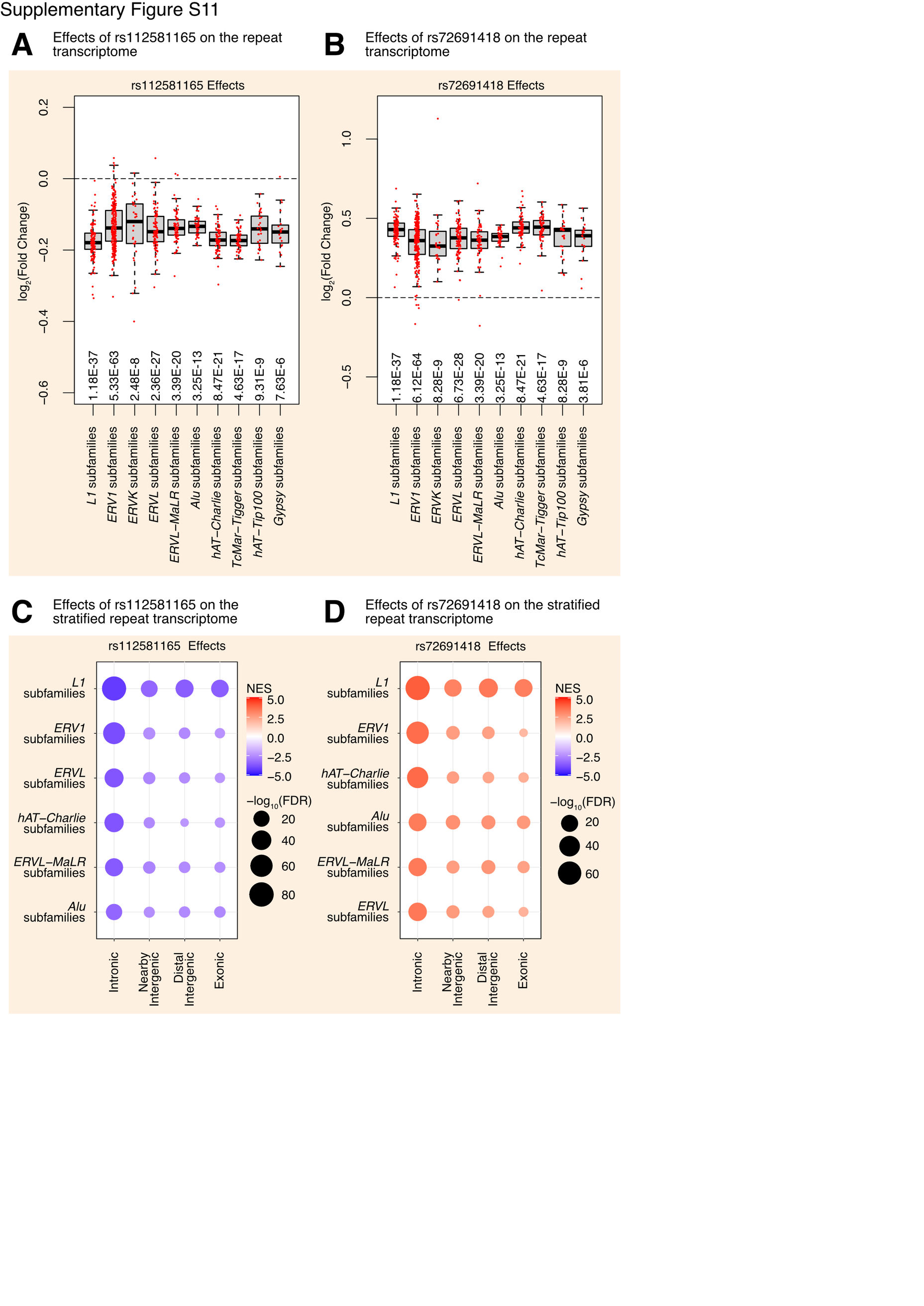

Supplement: S11 Fig — Box and whisker plots for the log2 fold changes of TE subfamilies (red dots), grouped by TE family, across genotypes for (A) rs112581165 and (B) rs72691418. A one-sample Wilcoxon test was run to determine whether changes were significantly different from 0. The FDR values from this test are listed at the bottom. GSEA analysis for top, differentially regulated TE family gene sets in different genomic regions (intronic, intergenic, exon-overlapping) across genotypes. The results for (C) rs112581165 and (D) rs72691418 are shown. In each bubble plot, the size of the dot represents the -log10(FDR) and the color reflects the normalized enrichment score. FDR: False Discovery Rate. (TIF) [file pgen.1011311.s011.tif]

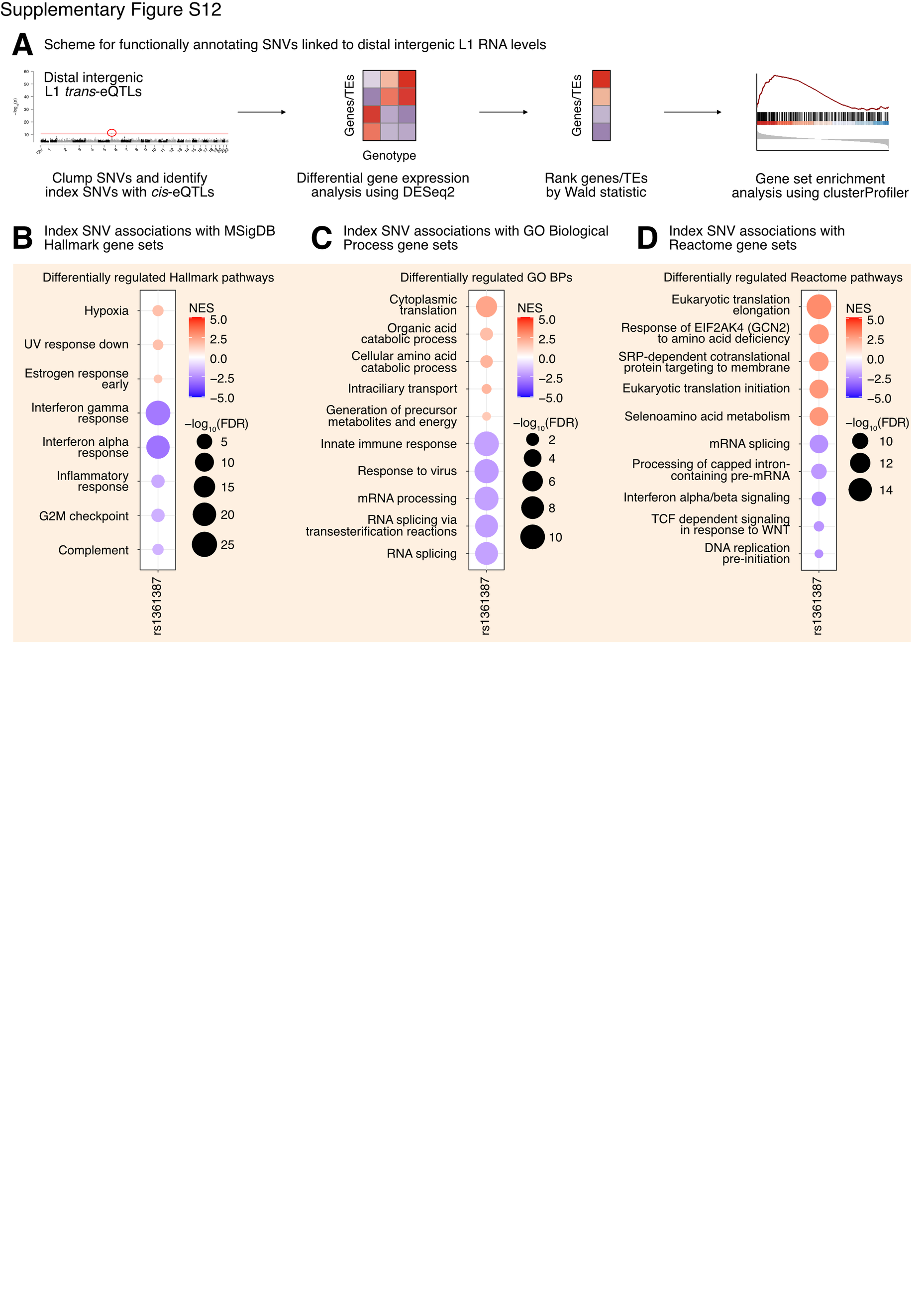

Supplement: S12 Fig — (A) Scheme for functionally annotating gene-linked index SNVs for distal intergenic L1 expression by GSEA. GSEA analysis for top regulated (B) MSigDB Hallmark pathway, (C) GO Biological Process, and (D) Reactome pathway gene sets across genotypes for rs1361387 (ZSCAN26). In each bubble plot, the size of the dot represents the -log10(FDR) and the color reflects the normalized enrichment score. FDR: False Discovery Rate. (TIF) [file pgen.1011311.s012.tif]

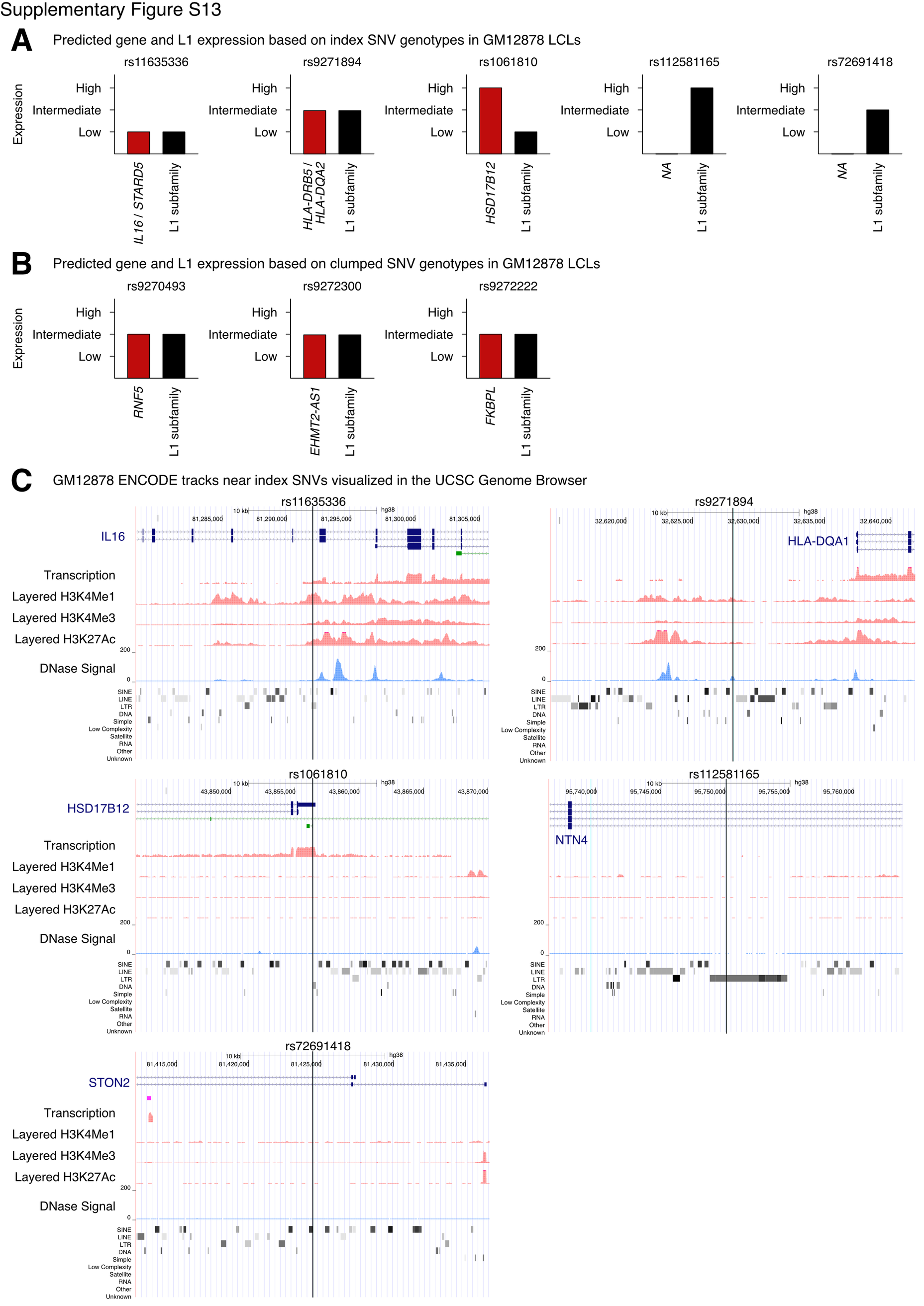

Supplement: S13 Fig — Though transcriptomic data is not available for these LCLs, the relative expression of candidate genes and linked L1 subfamilies can be predicted from GM12878 genotypes at either (A) index SNVs or (B) clumped SNVs. (C) ENCODE project epigenetic data available for GM12878 highlights regulatory markers near some trans-eQTL index SNVs. Data is visualized on the UCSC Genome Browser. (TIF) [file pgen.1011311.s013.tif]

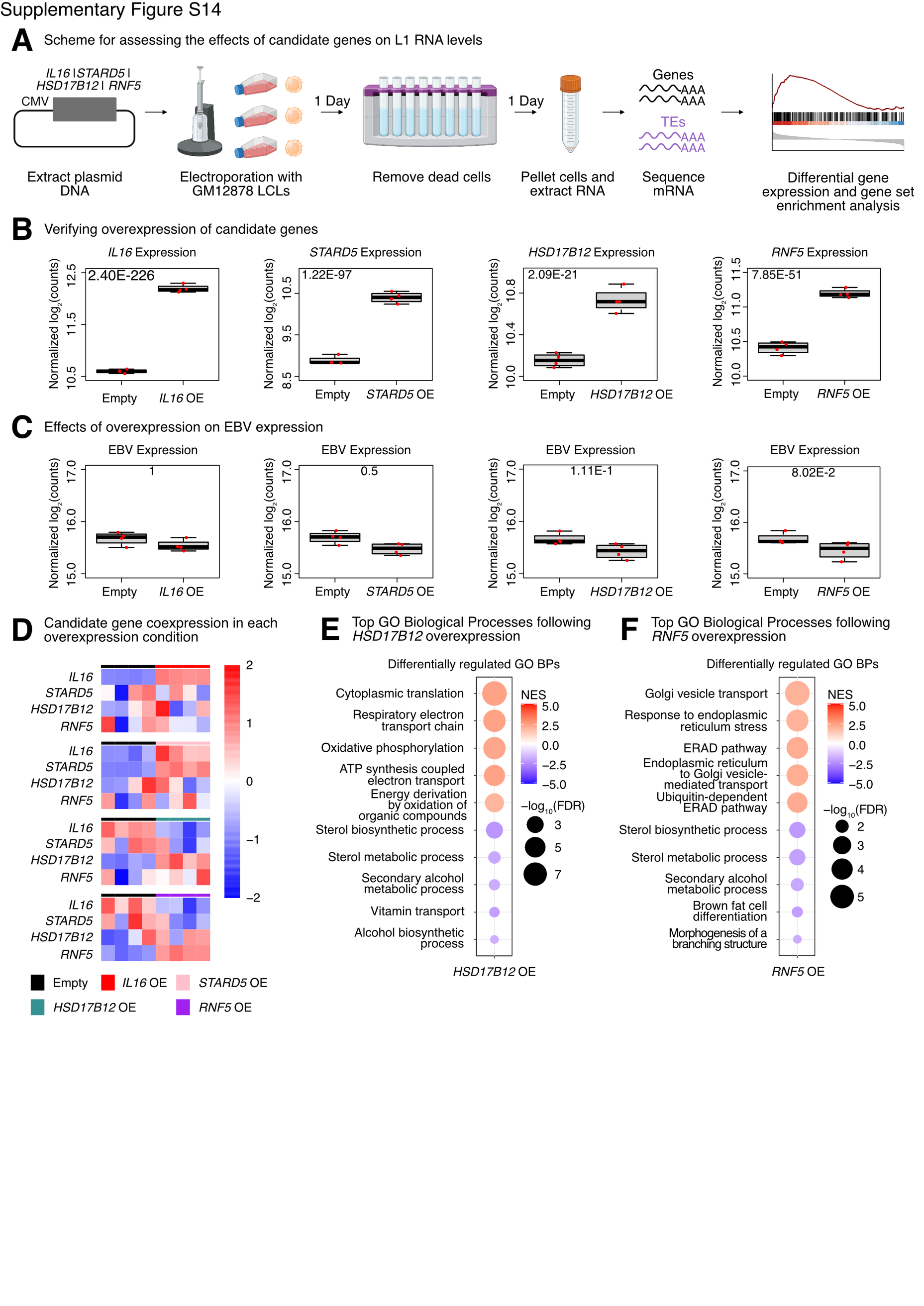

Supplement: S14 Fig — (A) Scheme for experimentally validating the roles of IL16, STARD5, HSD17B12, and RNF5 in L1 regulation. (B) VST-normalized log2 counts were quantified by DESeq2 for each gene being overexpressed. Each dot represents an independent transfection, with n = 4 per condition. The FDR for each comparison is listed at the top. (C) VST-normalized log2 counts for EBV were quantified by DESeq2 for each condition. Each dot represents an independent transfection, with n = 4 per condition. The FDR for each comparison is listed at the top. (D) Expression heatmaps for the four candidate genes tested, under each overexpression condition. GSEA analysis for top, differentially regulated (E) GO Biological Process gene sets following HSD17B12 overexpression. GSEA analysis for top, differentially regulated (F) GO Biological Process gene sets following RNF5 overexpression. In each bubble plot, the size of the dot represents the -log10(FDR) and the color reflects the normalized enrichment score. FDR: False Discovery Rate. Panel (A) was created with BioRender.com. (TIF) [file pgen.1011311.s014.tif]

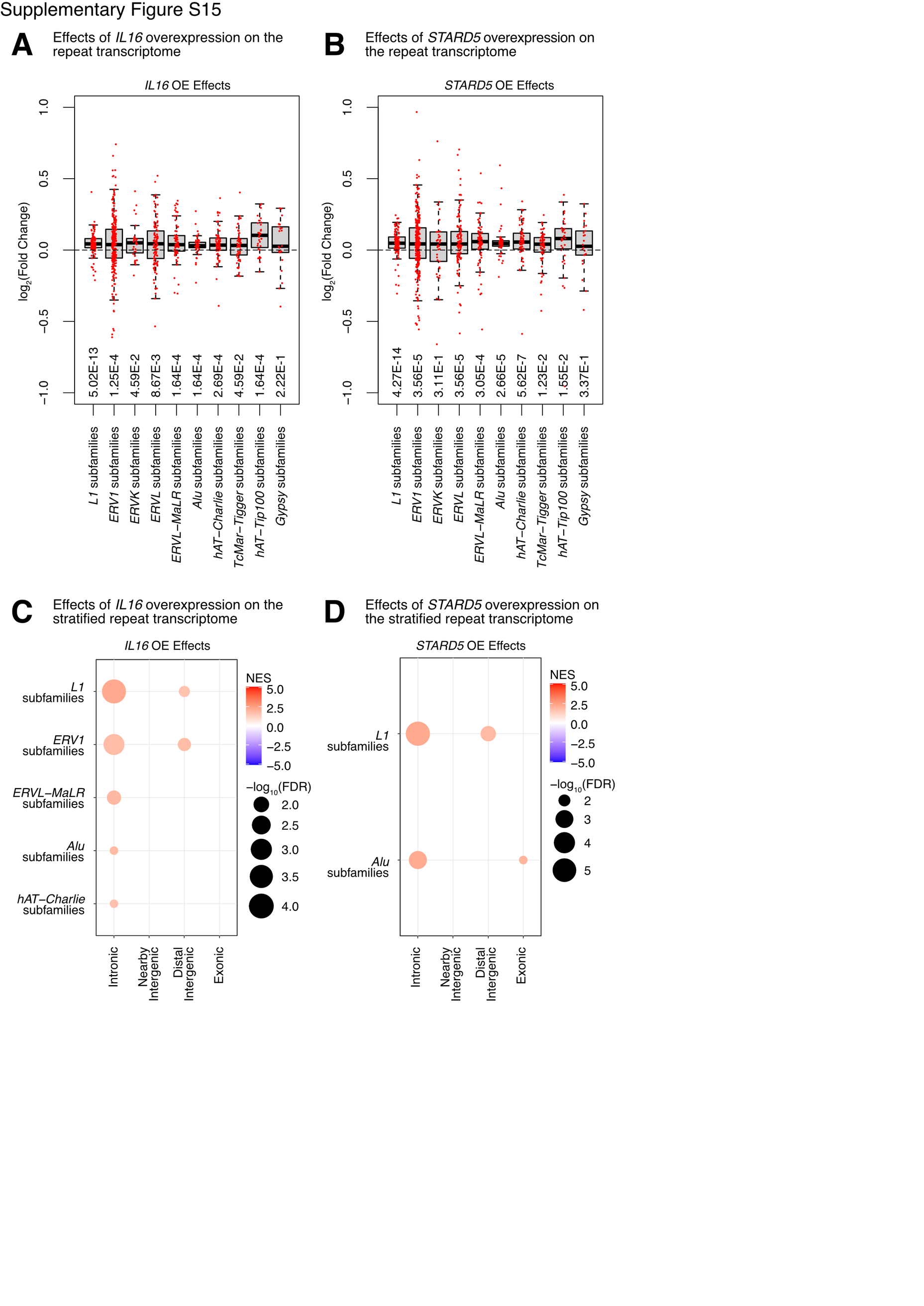

Supplement: S15 Fig — Box and whisker plots for the log2 fold changes of TE subfamilies (red dots), grouped by TE family, following (A) IL16 overexpression and (B) STARD5 overexpression. A one-sample Wilcoxon test was run to determine whether changes were significantly different from 0. The FDR values from this test are listed at the bottom. GSEA analysis for top, differentially regulated TE family gene sets in different genomic regions (intronic, intergenic, exon-overlapping) across overexpression condition. The results for (C) IL16 overexpression and (D) STARD5 overexpression are shown. In each bubble plot, the size of the dot represents the -log10(FDR) and the color reflects the normalized enrichment score. FDR: False Discovery Rate. (TIF) [file pgen.1011311.s015.tif]

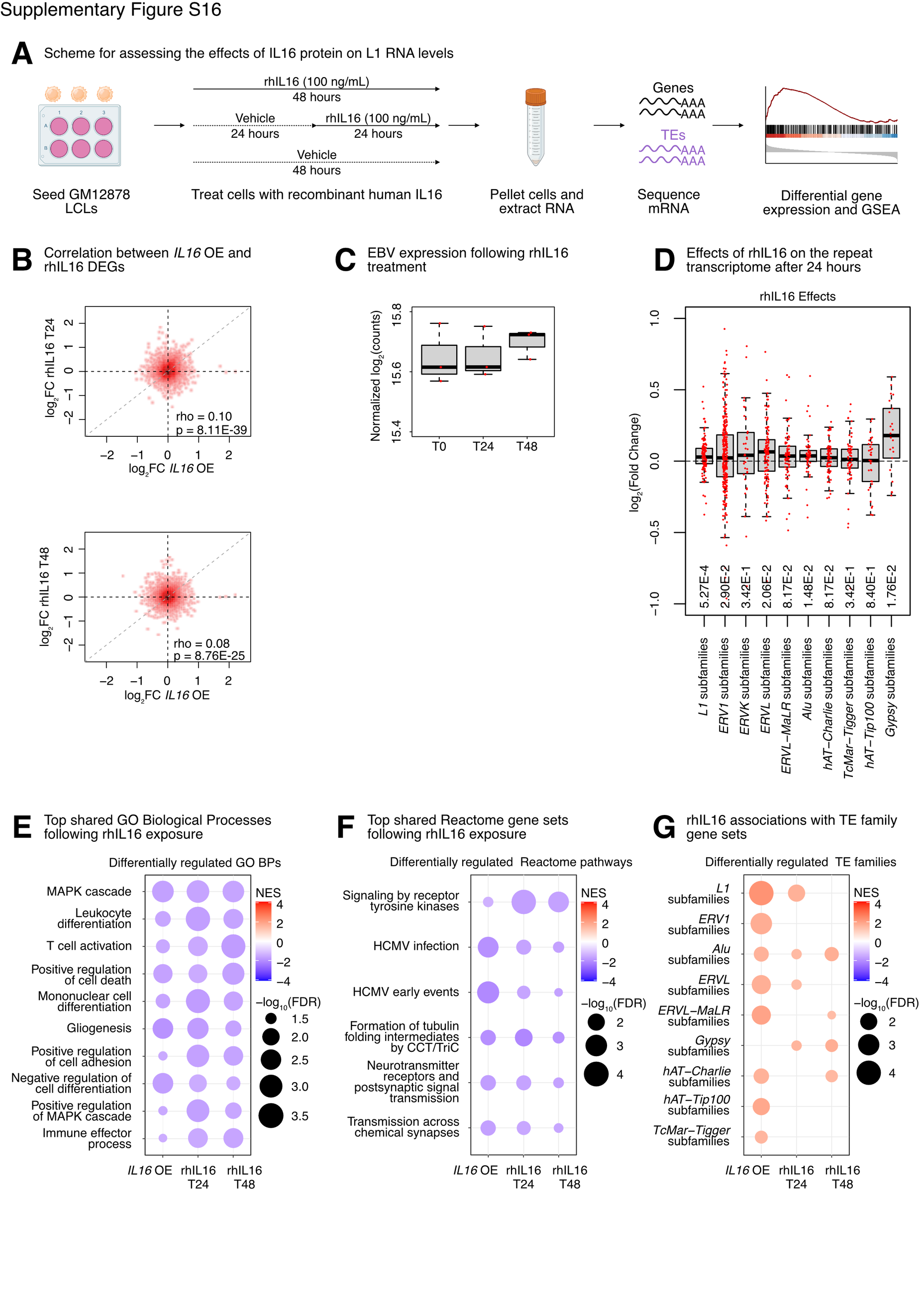

Supplement: S16 Fig — (A) Scheme for experimentally validating the role of rhIL16 in L1 regulation. (B) Spearman rank correlation between (top) 24-hour rhIL16 treatment differential expression or (bottom) 48-hour rhIL16 treatment differential expression and IL16 overexpression differential expression. (C) VST-normalized log2 counts for EBV were quantified by DESeq2 for each peptide treatment condition. Each dot represents an independent exposure, with n = 3 per condition. FDR > 0.05 at both the 24- and 48-hour treatment time points. (D) Box and whisker plots for the log2 fold changes of TE subfamilies (red dots) grouped by TE family after treatment with rhIL16 for 24 hours. A one-sample Wilcoxon test was run to determine whether changes were significantly different from 0. The FDR values from this test are listed at the bottom. GSEA analysis for top, shared, concomitantly regulated (E) GO Biological Process, (F) Reactome pathway, and (G) TE family gene sets following IL16 overexpression, rhIL16 exposure for 24 hours, and rhIL16 exposure for 48 hours. Shared gene sets were ranked by combining p-values from each individual treatment analysis using Fisher’s method. In each bubble plot, the size of the dot represents the -log10(FDR) and the color reflects the normalized enrichment score. FDR: False Discovery Rate. Panel (A) was created with BioRender.com. (TIF) [file pgen.1011311.s016.tif]

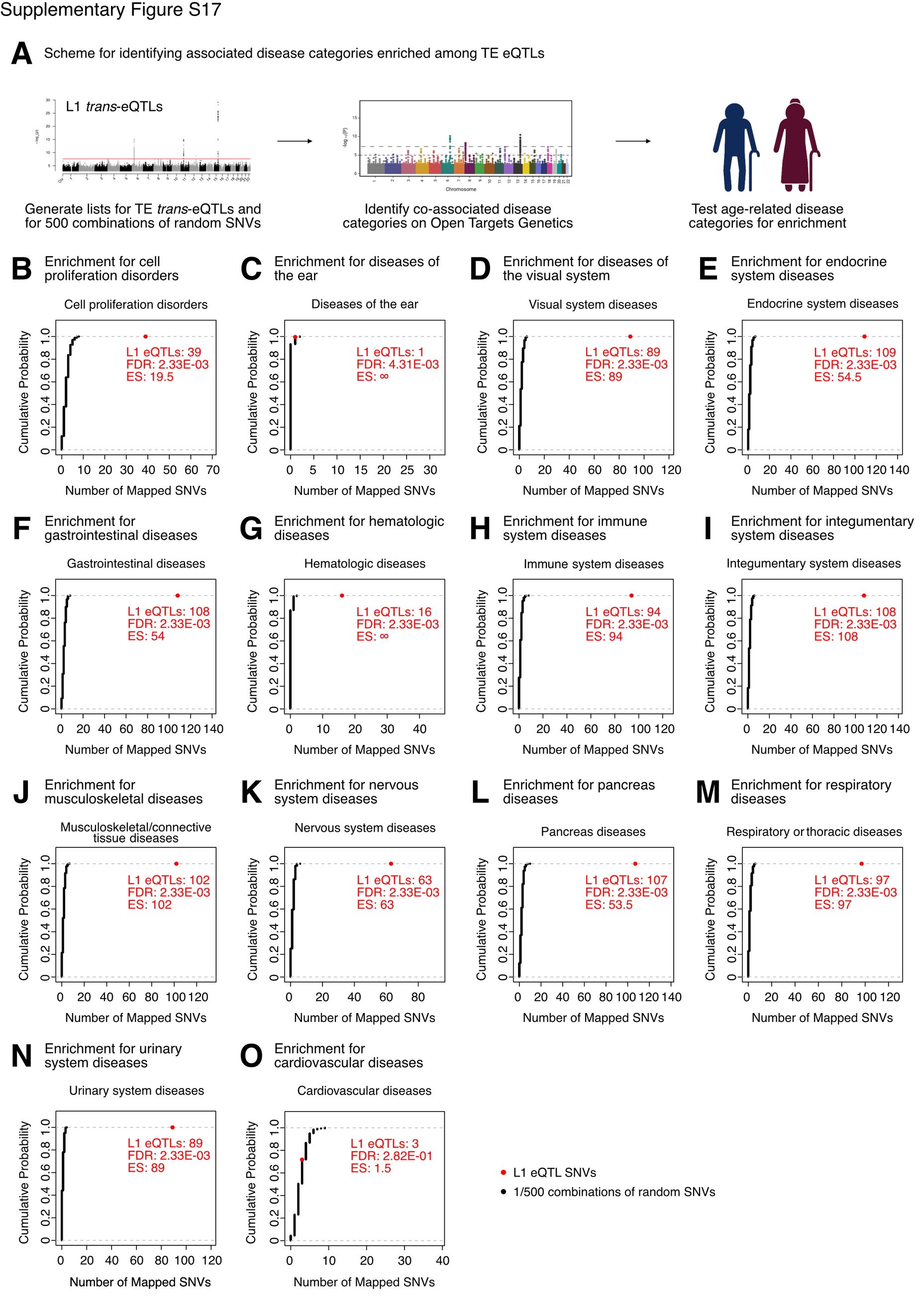

Supplement: S17 Fig — (A) Scheme for assessing enrichment of disease category associations among L1 trans-eQTLs. L1 eQTLs or combinations of random SNVs were queried on the Open Targets Genetics platform, and the number of SNVs mapping to 14 disease categories annotated by the platform were calculated. An empirical cumulative distribution function (ecdf) was defined for each disease category using the associations for the random SNVs, and this function was used to calculate an enrichment p-value, defined as p = 1 –ecdf(mapped eQTLs). Afterwards, p-values were FDR-corrected. An enrichment score (ES) was calculated by taking the number of mapped L1 eQTLs for a category and dividing by the median number of mapped SNVs among the random SNV combinations. Categories with an ES > 1 and FDR < 0.05 were considered significantly enriched. The number of mapped SNVs relative to the cumulative probabilities from the simulations are shown for (B) cell proliferation disorders, (C) diseases of the ear, (D) diseases of the visual system, (E) endocrine system diseases, (F) gastrointestinal diseases, (G) hematologic diseases, (H) immune system diseases, (I) integumentary system diseases, (J) musculoskeletal diseases, (K) nervous system diseases, (L) pancreas diseases, (M) respiratory diseases, (N) urinary system diseases, and (O) cardiovascular diseases. FDR: False Discovery Rate. Panel (A) was created with BioRender.com. (TIF) [file pgen.1011311.s017.tif]
