# Supplementary material for: An eQTL-based approach reveals candidate regulators of LINE-1 RNA levels in lymphoblastoid cells
Source: PLoS Genet. 2024 Jun 7;20(6):e1011311. doi: 10.1371/journal.pgen.1011311 (PMC11189215; doi:10.1371/journal.pgen.1011311)
Supplement: Attachment — Submitted filename: 139998_1_rebuttal_2864496_s4wdd8.docx [file pgen.1011311.s027.docx]

**Manuscript number:** RC-2023-02167

**Corresponding author(s):** Bérénice, Benayoun A

## 1. General Statements

We thank the reviewers for providing feedback that we think has helped expand and clarify our findings. Importantly, we note that the ultimate goal of this study is to identify candidate regulators of L1 RNA levels, which are influenced by both transcriptional and post-transcriptional processes (such as RNA targeting by miRNAs or alternative splicing and incorporation of intronic TEs into mature mRNAs). Due to our initial and loose use of the term ‘transcriptional regulator’, we gave the impression that were looking for transcription factors or other elements that bind the L1 internal promoter to activate L1 transcription. Though this was of interest, this was only a subset of the types of interactions we were scanning for. We have amended the manuscript to describe our search for more general regulators of L1 RNA levels, and we apologize for the confusion.

Importantly, although increasing evidence is revealing that non autonomous L1 elements still have biologically significant regulatory and functional potential, the control mechanisms and functional consequences of L1 RNA fragments or non-autonomous L1 copies remains incomplete. For example, one study suggests that intronic L1s are part of an important regulatory network maintaining T-cell quiescence (Marasca et al. 2022). This is consistent with the increasing appreciation for the importance of alternative splicing in immune regulation and cell death pathways (Liao and Garcia-Blanco 2021) and with another study highlighting the importance of TE exonization in interferon signaling (Pasquesi et al. 2023). Additionally, L1 RNA may be sufficient to induce an interferon response and alter cellular viability, even in the absence of transposition (Ardeljan et al. 2020; Luqman-Fatah et al. 2023). Given these observations, there is a need to characterize the control mechanisms and functional effects of all L1 RNA sources, including both intronic and intergenic copies.

Finally, we acknowledge that, in agreement with reviewers’ comments, regulatory programs and mechanisms may be specific to intronic, exonic, or intergenic L1s. Thus, in this revised manuscript, we have also replicated all of our analyses using TE profiles stratified by genomic context (intronic, distal intergenic, nearby intergenic, and exon-overlapping). This stratification allowed us to (i) identify new SNVs associated with each of these RNA profiles and (ii) demonstrate that our treatments (*IL16* overexpression, *STARD5* overexpression, and rhIL16 treatment for 24 hours) influence both intronic and distal intergenic L1 RNA levels. *These new findings, together with the previously described enrichment of aging and aging-related SNVs, suggest to us that our findings are functionally relevant for transposon and aging biology.*

## 2. Point-by-point description of the revisions

**Reviewer #1 (Evidence, reproducibility and clarity (Required)):**

 The work by Bravo and colleagues describes a novel and interesting study aimed at identifying potential transcriptional and regulatory interactions between transposable elements (TEs), SNs/SNVs and TEs/genes expression exploiting public data from the 1000 Genomes and the Geuvadis consortia. The study is well designed, described and developed. The ideas are rather novel and interesting despite the application of rigorous and well established analytical tools and strategies. The results are robust and convincing, and the figures and supplementary materials are extremely clear and complete. The authors also validate using cell cultures some of their findings which make the article and its results more interesting and valuable.

We thank the reviewer for their positive words and evaluation of our study and its significance.

I suggest to expand the introduction and discussion. In the introduction I suggest to refer to previous works which prepared the way for this article such as https://pubmed.ncbi.nlm.nih.gov/31757210 and https://pubmed.ncbi.nlm.nih.gov/28824558.

We agree that these landmark papers should be highlighted, as they provide important context for the use of the GEUVADIS and 1000Genomes data in studying transposon regulatory mechanisms. Consistently, we have added the following to the text in our revised manuscript (page 5, line 123): “Later, in a series of landmark studies on TE biology, this collection of data was used to (i) characterize the effects of polymorphic TE structural variation on gene expression (TE-eQTLs) (Wang et al. 2016; Spirito et al. 2019; Goubert et al. 2020) and (ii) explore the potential impact of TE polymorphisms on human health and disease through genome-wide association study (GWAS) analysis (Wang et al. 2017). These results highlight the value of this data and the power of eQTL analysis in identifying genetic factors implicated in gene expression control and, potentially, disease susceptibility.”

Regarding the Discussion it would be useful to discuss possible influences of polymorphisms and specify whether the identified regulators contain in their genomic loci a full length L1 element which might be at the basis of the observed relationships.

We thank the reviewer for their suggestion. We were curious about this as well, and we reasoned that polymorphic L1s would vary with the hosting gene and would thus have been identified by Wang et al. 2016 in their TE-eQTL study. However, the top candidates we tested—*IL16, STARD5, HSD17B12, and RNF5*—were not associated with L1 polymorphisms, to our knowledge. Thus, it is likely that a polymorphic L1 would be a trivial explanation for the described regulatory relationships. We have added the following to the discussion section (Page 25, line 719): “To note, none of our top candidates were associated with L1 polymorphisms in the landmark TE-eQTL study that first used this data to study TE biology (Wang et al. 2016), suggesting that our findings are likely mechanistically independent.”

****Referees cross-commenting****

I generally agree with the doubts expressed by the other 2 referees. The main raised problems relate to a poor inspection and characterization of analyzed L1s expression and genomic structure. The 2 major highlighted potential issues are 1) co-transcription of L1s present in loci were the real target of the putative regulator is another canonical gene and 2) transcription of exonized L1 fragments as part of other transcriptional unit independent by TEs. The issue 1) is a general problem of trans-eQTL, however this not raise the danger to select false positives, so to me it would be sufficient to stress the issue in the discussion. The issue 2) is more important and can cause the identification of false positive if not properly addressed. One solution might be to select and study only full length intergenic elements taking advantage, for example, of the annotations from L1base [https://pubmed.ncbi.nlm.nih.gov/27924012](https://urldefense.com/v3/__https:/pubmed.ncbi.nlm.nih.gov/27924012__;!!LIr3w8kk_Xxm!oHqr6LfSmnjKU0vpJjnrJ8nJ9ESdNYEatzFZeeFi7DRTCsPt1GyV-mvJSF44kA74QUl9HHJyQKkcgMJ0RUCBOH8$)/

The reviewer’s point are well taken, and we apologize for using imprecise language in the first version of this manuscript, using “transcription” as a shortcut to encompass all mechanisms that regulate RNA levels (including promoter-dependent transcription, mRNA turnover, intron retention, *etc*.). As highlighted in our general statement above, we note that any phenomenon regulating the levels of TE-derived RNA in cells can lead to clear biological consequences without the need for transposition or promoter-driven transcription [Marasca et al. 2022; Pasquesi et al. 2023; Ardeljan et al. 2020; Luqman-Fatah et al. 2023]. We have amended our language throughout the revised manuscript to make this point clear. Indeed, the analysis of association of the identified SNPs with regards to aging/age-related diseases (**Figure 7**, **Supplemental Figure S17**) shows that the identified SNPs are biologically relevant, much above what is expected for any random subsample of SNPs.

The initial goal of our study was to identify candidate genes that altered repetitive RNA levels irrespective of genomic location (intergenic, intronic, or exonic). We sought to do this on the basis that repetitive RNA alone, even in the absence of transposition and even if originating from intronic loci or as part of an exonization event, can exert biologically meaningful effects [Marasca et al. 2022; Pasquesi et al. 2023; Ardeljan et al. 2020; Luqman-Fatah et al. 2023]. Moreover, an analysis of TE expression in tumor-adjacent tissue [Chung *et al*., 2019] observed prevalent co-expression of different TE subfamilies and included both intronic and intergenic TEs, suggesting that minor alterations in TE expression can affect global TE expression profiles. Indeed, gene set enrichment analysis (GSEA) using TE family gene sets shows global but subtle alterations in TE expression in response to eQTL genotypes and in response to our treatments. We now discuss these studies to add additional context to our goals and our analysis results (Page 6, Line 145; Page 13, Line 354).

However, we do agree with the reviewer that it is biologically significant to delineate the contributions of different TE genomic loci (intergenic, intronic, exonic) to the repetitive RNA pools. Consequently, we obtained locus-level TE quantifications using the TElocal package (a companion tool to TEtranscripts) and aggregated TE expression depending on the following criteria: (i) intergenic TEs farther than 5 kb from a gene were considered ‘distal intergenic TEs’, (ii) intergenic TEs within 5 kb of a gene were considered ‘nearby intergenic TEs’, (iii) TEs overlapping any annotated exon GENCODE33 were considered potentially ‘exonic TEs’, and (iv) TEs not overlapping any annotated exon and falling within a gene were considered ‘intronic TEs’. We repeated our eQTL analysis and all downstream gene set enrichment analyses (GSEA) using these 4 updated categories (i) to characterize the genomic architecture contributing to RNA level differences in each type of TE category and (ii) to characterize the effects of our treatments on each TE category. Finally, we have updated the introduction section to highlight our rationale for analyzing repeats across the four categories (Page 6, Line 145), and we have updated our discussion section (Page 25, line 723; Page 26, line 734) to incorporate our findings across the four categories.

**Reviewer #1 (Significance (Required)):**

The strength of the work rely mainly in the biological validations, while its limitation can be addressed in the relatively limited sample coverage. The identification of potential L1 regulators using the strategy presented by the author is a novel and sound approach which can result of interest to a broad audience. I am a molecular and computational biologist interested in transposable elements function, regulation and evolution.

We thank the reviewer for highlighting the interest and novelty of our work.

**Reviewer #2 (Evidence, reproducibility and clarity (Required)):**

Summary:

The manuscript by Bravo et al. aims at identifying regulators L1 retrotransposon expression using an eQTL approach taking advantage of paired RNA-seq and whole genome sequencing data generated by the 1KGP and the Geuvadis consortia. The general idea is to identify trans-eQTL that could be associated with a change of L1 expression in lymphoblastoid cell lines.

We thank the reviewer for their careful evaluation of our methodology. We believe their comments significantly enhanced our study.

Major comments:

1. The overall strategy for the quantification of L1 expression is flawed and unlikely to bring any meaningful insight. The reason is that the authors use aggregated transposable element (TE) expression. As many TEs are inserted in genes, a significant fraction of the measured TE expression signal originates from cotranscription with genes or from pervasive transcription, even for mRNA sequencing, which selects poly(A)+ RNA (see Deininger et al. NAR 2017, Navarro et al. PLoS Comp Biol 2019, Faulkner Neuron 2023; reviewed in Lanciano et al. Nat Rev Genet 2020). As a consequence, it is impossible to conclude whether apparent variations of TE expression result from changes of TE transcription itself (i.e. from the TE promoter) or from changes of expression of genes containing this family of TEs.

We understand the concerns raised by the reviewer and believe that part of the perceived issue was from our imprecise use of “transcription” in our initial submission. We apologize for this imprecision, where we loosely used “transcriptional levels” (implying promoter-dependent transcriptional regulation as a mechanism) as a shortcut for “RNA levels” (encompassing any and all mechanisms altering cellular levels of an RNA-species, including – but not exclusively – resulting from promoter-dependent transcription, RNA turnover, retained intron, etc.).

As highlighted in our general statement, the central goal of our study was to identify candidate genes that altered transposon RNA levels, irrespective of genomic location (intergenic, intronic, or exonic). We sought to do this on the basis that transposon RNA alone, even in the absence of transposition, if originating from intronic loci or even as part of an exonization event, can exert biologically meaningful effects [Marasca et al. 2022; Pasquesi et al. 2023; Ardeljan et al. 2020; Luqman-Fatah et al. 2023]. Moreover, a published analysis of TE levels in tumor-adjacent tissue [Chung *et al*., 2019] observed prevalent co-expression of different TE subfamilies, suggesting that minor alterations in TE levels can affect global TE expression profiles. Consistently, our gene set enrichment analysis (GSEA) using TE family gene sets shows global but subtle alterations in TE family levels in response to eQTL genotypes and in response to our treatments.

However, we do agree that it is meaningful to delineate the contributions of different TE genomic locations (intergenic, intronic, exonic) to the repetitive RNA pools. Consequently, as suggested by the reviewer, we obtained locus-level TE quantifications using the TElocal package (a companion tool to TETranscripts) and aggregated TE expression depending on the following criterial: (i) intergenic TEs farther than 5 kb from a gene were considered ‘distal intergenic TEs’, (ii) intergenic TEs within 5 kb to a gene were considered ‘nearby intergenic TEs’, (iii) TEs overlapping any annotated exon were considered potentially ‘exonic TEs’, and (iv) TEs not overlapping any annotated exon and falling within a gene were considered ‘intronic TEs’.

Using this stratified data, we have now repeated our eQTL analysis (**Supplementary Figure S7**) and all downstream gene set enrichment analyses (GSEA) (**Supplementary Figure S9D-F, S11C-D, S15C-D** and **Figure 5F**) using these updated categories (i) to characterize the genomic architecture contributing to RNA level differences in each type of TE category, and (ii) to characterize the effects of our treatments on each TE category. Finally, we have updated the introduction section to highlight our rationale for analyzing repeats across the four categories (Page 6, Line 145), and we have updated our discussion section to incorporate our findings across the four categories (Page 25, line 723; Page 26, line 734).

Interestingly, using this complementary approach for the eQTL analysis, we find that many of our “global” hits are recapitulated across the 4 categories (new **Figure S7**). Interestingly, we also identified new potential regulators in the stratified approach, including *ZSCAN26* (new **Figure S7C; Figure S8; Figure S12B**), a zinc finger and SCAN domain containing protein, which may be another promising regulator. Although we do not follow up on this candidate in the present manuscript (as we believe it is outside of the scope of this study), we believe that the additional targets identified will be useful for the community. We discuss the common and divergent results from both approaches in the revised manuscript (Page 11, line 303).

To illustrate this point, a quick search indicates that one of the top hits, IL16, proposed to regulate L1P4a expression, contains an intronic copy of a fragmented L1P4a element. Given the absence of changes in L1P4a expression upon IL16 overexpression or recombinant IL16 treatment, it appears that the most likely scenario relates to pervasive transcription. Note that the incriminated L1 element does not have to be inserted in the candidate gene, but can also be close to it, or even located in another gene regulated by the candidate gene.

We thank the reviewer for highlighting this possibility. Indeed, one of the motivations for constructing TE family gene sets and carrying out the secondary GSEA was to identify alterations in repeats beyond the lone repeat subfamily identified in the eQTL analysis. In the initial manuscript, we could not determine the source of these global changes (i.e. intronic/exonic/intergenic). Therefore, we have repeated the GSEA analyses using repeat subfamily expression profiles stratified by these categories. Importantly, our new analysis shows that our top candidate regulators *IL16/STARD5*, although originally identified in the eQTL analysis as part of the “intronic TE” hits, exert significant effect on the levels of distal intergenic TEs as well (new **Figure S15C, D** and **Figure 5F**). We now discuss these new findings in the text (Page 20 line 577; Page 21 line 609). We thank the reviewer for suggesting this analysis that brought additional insights to our work.

2. All downstream analyses are impacted by the issue described above.

We agree that the initial approach did not take into consideration the distinction between TEs transcribed as part of a gene unit and those transcribed from their own promoter. As highlighted above, we now include a new stratified analysis of TE RNA level profiles into intronic/exonic/intergenic categories.

Specific to the reviewer’s concern, TEs in the distal intergenic category (>5 kb from a gene) should not produce RNA that is co-transcribed with a gene or arising as part of an exonization event. We have repeated our eQTL and GSEA analyses using these stratified expression profiles. Importantly, we note that our top hits, *IL16/STARD5*, exert significant effect even on the levels of distal intergenic TEs as well (new **Figure S15C, D** and **Figure 5F**), suggesting that the observed effects are not the mere result of nearby regulation of bystander TE transcription, as originally feared by the reviewer.

Moreover, even if the issue of co-transcription with genes could be solved, I doubt that performing GSEA (and probably other types of GO-term analyses) on TE families with various copy number could be a valid approach.

Although we do understand the reviewer’s concern, we note that, despite the expressed doubts, GSEA was specifically designed as an algorithm to identify subtle consistent effects on gene categories (given that traditional overrepresentation analyses approaches tend to be underpowered and produce many false negative results) [(Subramanian et al. 2005]. Indeed, we are not the first ones to use this approach, which has been broadly validated by over a decade of computational biology analyses.

Specifically, GSEA was developed in response to (i) the lack of reproducibility of individual, significant gene changes across studies (especially due to varying number of replicates and thus power to detect differential genes robustly) and (ii) the need to summarize biological changes in a functionally meaningful way through the use of gene sets containing biologically-related genes. Often, sets of genes sharing a molecular function (MF), cellular component (CC), or biological process (BP) derived from the Gene Ontology (GO) project are used for this purpose (and indeed we used these types of gene sets to identify biological processes correlated with TE expression differences).

However, any gene set can be analyzed in this fashion as the algorithm is agnostic to the nature of the used gene sets - it can include any set of functionally related elements. For the TEs, we opted to aggregate TE subfamilies into gene sets corresponding to TE families on the basis that (i) broad changes in individual TE subfamilies may be hard to detect but changes across many subfamilies would be easier to detect, (ii) the expression of many different TE subfamilies was previously found to be highly correlated in an analysis of tumor-adjacent tissue [Chung *et al*., 2019], (iii) we were searching for factors influencing global TE RNA levels and not just specific TE loci, and (iv) GSEA has previously been applied to summarize TE changes [Gu et al. 2021; Wong et al. 2021; Zhang et al. 2023]. Thus, we respectfully disagree with the reviewer’s intuition about GSEA’s appropriateness in the case of TE-related analyses. To make our reasoning and the aim of the GSEA analysis clearer, we now explicitly discuss these points in the revised manuscript (page 13, line 348).

In addition, to provide an orthogonal/independent approach to the GSEA analysis that should alleviate the reviewer’s concerns, we also now include an alternate non GSEA-based approach to determine potential widespread effects on TE family RNA levels, testing whether the distribution of log2(Fold Change) in levels are significantly different to 0 (*i.e*. no change in TE RNA level) using the non-parametric Wilcoxon test (new **Supplementary Figure S9A-C, S11A-B, S15A-B, S16D).** The results are consistent with our GSEA findings, validating the presence of widespread significant effects on TE family levels.

Finally, we have also applied the GSEA approach to the intergenic/intronic/exonic TE expression profiles to better assess the functional significance of the identified L1 eQTLs and treatments as a function of TE genomic context.

We believe the additional clarifications and new analyses should help alleviate the concern that our results are an artefact of the GSEA method.

Minor comments:

3. The literature linking aging and L1 is extrapolated. In the abstract, l29-31, 'established' and 'widely recognized' are very strong statements. P.3, the original findings of increased L1 copy number in senescent cells (ref 11-12) were later shown by the same group that these extra copies were likely extra-chromosomal and cytosolic (ref 15).

We apologize for potentially extrapolating some of the published data in the field. We have rewritten these findings in a more conservative tone, as suggested by the reviewer (Page 2, line 31); Page 3, line 68).

4. l.55: only L1Hs are retrotransposition-competent.

We thank the reviewer for bringing this typo to our attention. It has been corrected.

5. l.77: typo at "normally silenced".

We thank the reviewer for bringing this typo to our attention. It has been corrected.

6. Fig. 1B: it is unclear what is illustrated by the panels at top and bottom right.

We apologize for our lack of clarity in the original submission. The diagram was meant to illustrate the main output of the eQTL analysis: genes that were positively correlated with TE expression as the genotype was alternated (top; which may represent, for example, positive regulators of TE expression) and genes that were negatively correlated with TE expression as the genotype was alternated (bottom; which may represent, for example, repressors of TE expression). We have added “Potential L1 activators” and “Potential L1 repressors” labels to the figure to clarify.

****Referees cross-commenting****

I agree that focusing only on intergenic full length elements could mitigate the issues that were raised (which apply to both exonic and intronic elements). However, this would necessitate to quantify TE expression at the locus-level, rather than to aggregate TE expression at the family level. For the youngest elements, such as L1Hs, this could actually a major technical difficulty. In general, it implies that all the analyses performed in the paper - and likely the experimental validations - should be repeated.

First, as discussed in the general statement, we want to note that much of the issues raised by the reviewer stem from the understanding that we were looking exclusively for promoter-dependent effects on TE levels. Again, we note that in many instances, TE RNA can lead to clear biological consequences without the need for transposition or promoter-driven transcription [Marasca et al. 2022; Pasquesi et al. 2023; Ardeljan et al. 2020; Luqman-Fatah et al. 2023]. We have amended our language to make it clear that our interest lies in TE RNA levels, rather than specific transcriptional regulation throughout the revised manuscript.

However, we agree with the reviewers that additional granularity in the analysis may provide important additional insights into TE biology. As noted, the technical difficulty in obtaining locus-level quantifications for younger elements like L1Hs is one of the many reasons why we opted for a *trans*-eQTL approach at the subfamily level. Importantly, other key reasons were that (i) it would require thousands of samples to be powered for genome-wide locus-specific TE QTL analysis rather than trans-eQTL, and (ii) we were looking for global regulators, not regulators of specific copies (which would be less likely to exert broad biological effects).

As highlighted above, using an aggregate, *trans*-eQTL scan avoids the need for locus-specific quantification and provides greater detection power for broad biological effects. However, the issues regarding specificity of exonic/intronic repeats remain. Consequently, as described in our responses to the above points made by the reviewer, we now include analyses of repeat levels stratified by genomic context (intronic/exonic/intergenic) and aggregated at the subfamily level (**Supplementary Figure S7, S9D-F, S11C-D, S15C-D** and **Figure 5F**).

Using these stratified repeat expression profiles, we have repeated all the analyses performed in the paper. Importantly, our main conclusions stand, including by showing that even candidate regulators identified in the “intronic“ hits can exert effect on distal intergenic elements (new **Figure S15C, D** and **Figure 5F**).

**Reviewer #2 (Significance (Required)):**

The study is timely as it could in principle nicely complements published functional genomic screens (Liu et al. Nature 2018; Sun et al. PNAS 2018; Mita et al. NSMB 2020; Briggs et al. Mob DNA 2021, etc), but as underlined above, the described work is unconclusive, and the effects detected are very "subtle", as the authors themselves point out. My field of expertise relates to molecular and cellular biology and genomics, and my works has a focus on transposable elements.

We thank the reviewer for recognizing the timeliness of our study. In addition, we reiterate that the aim of our study was to develop a pipeline that complements (not replaces) existing screening methods, all of which have their own set of limitations. Though the effects we highlighted may be subtle, such effects are not completely unexpected in healthy, natural populations without obvious pathology. Specifically, individuals harboring genetic variants that grossly regulate TE expression levels would be strongly negatively selected due to deleterious genomic instability and/or inflammation. This is in contrast to *in vitro* screening systems where inducing high TE expression in cell lines that can tolerate high TE expression is the norm (as in Liu et al. Nature 2018 and Mita et al. NSMB 2020). Moreover, though the effects may be subtle, that does not imply that the effects are functionally meaningless: indeed, the genetic variants underlying these subtle differences are significantly enriched for co-association with aging and age-related disease phenotypes in GWAS databases. Thus, we believe that the ability to detect subtle TE expression differences in natural, healthy populations is actually a strength of this new approach, with such regulators more likely to exert physiological functions in healthy human rather than screening artefacts.

**Reviewer #3 (Evidence, reproducibility and clarity (Required)):**

In their manuscript "An eQTL-based Approach Reveals Candidate L1 Transcriptional Regulators in Lymphoblastoid Cells," Bravo et al. describe their approach for identifying genes associated with regulation of transposable element expression. While the title suggests the analyses of factors that are associated with L1 expression, the manuscript has a broader scope, and indicates the presence of transposable element expression of numerous different transposable element families. The authors pursue several different approaches to gain deeper insights into their findings. I have been impressed by the breath of the analyses. While the sample size and population diversity may be smaller than desired, the publicly available datasets are responsible for this limitation.

We thank the reviewer for their positive outlook on our work, and share their desire for larger, more diverse datasets for further applications of our approach.

My primary concern relates to main study objective: L1 or transposable element expression. Based on the information Bravo et al. provide, unfortunately, I am not convinced that the authors observed canonical L1 or transposable element expression. To the research community's understanding, transposable element expression should be skewed toward the youngest subfamilies. However, based on the text, figures, and supplemental data, transposable element expression seemed to be enriched for old subfamilies. Several of the observed transposable element families have ceased activity for tens of millions of years. The observed patterns seem to suggest that the transposable sequences are within (m)RNA sequences and represent exonization events. The authors do not provide clear evidence for either transposable element expression that initiates at the transposable element sequence and utilizes their promoter or exaptation of transposable element sequences. (Some of the references engage with both.)

We thank the reviewer for raising these concerns. As discussed in our general statement, we believe that our loose use of “expression/transcription” wrongly implied that our study was exclusively interested in identifying promoter-dependent regulation of TE transcription, when we were interested in any regulators altering cellular levels of TE-derived RNA, including – but not exclusively – resulting from promoter-dependent transcription, RNA turnover, retained intron, etc. We sought to do this on the basis that transposon RNA alone, even in the absence of transposition, if originating from intronic loci or even as part of an exonization event, can exert biologically meaningful effects [Marasca et al. 2022; Pasquesi et al. 2023; Ardeljan et al. 2020; Luqman-Fatah et al. 2023]. Moreover, a published analysis of TE level in tumor-adjacent tissue [Chung *et al*., 2019] observed prevalent co-expression of different TE subfamilies, suggesting that minor alterations in TE levels can affect global TE expression profiles. Consistently, our gene set enrichment analysis (GSEA) using TE family gene sets shows global but subtle alterations in TE family levels in response to eQTL genotypes and in response to our treatments. We apologize for the confusion, and have updated the manuscript, including in the title and introduction (Page 6, line 132) to clarify the purpose of the study.

However, we recognize that important biological insights can be gained by delineating the contributions of different TE genomic locations (intergenic, intronic, exonic) to the repetitive RNA pools. Consequently, in this revised manuscript, we obtained locus-level TE quantifications and aggregated TE levels depending on the following criteria: (i) intergenic TEs farther than 5 kb from a gene were considered ‘distal intergenic TEs’, (ii) intergenic TEs within 5 kb to a gene were considered ‘nearby intergenic TEs’, (iii) TEs overlapping any annotated exon were considered potentially ‘exonic TEs’, and (iv) TEs not overlapping any annotated exon and falling within a gene were considered ‘intronic TEs’. We have repeated our eQTL analysis (**Supplementary Figure S7**) and all downstream gene set enrichment analyses (GSEA) (**Supplementary Figure S9D-F, S11C-D, S15C-D** and **Figure 5F**) using these updated categories (i) to characterize the genomic architecture contributing to RNA level differences in each type of TE category and (ii) to characterize the effects of our treatments on each TE category.

Additionally, we have carried out additional GSEA analyses using gene sets of L1 subfamilies split by evolutionary age (i.e. L1M, L1P, and L1PA) to assess their responses to our treatments (**Figure 3D, 4H, 5E**).

We hope that these additional clarifications and analyses alleviate the reviewer’s concerns.

Furthermore, the patterns of gene activation seem to be in alignment with viral EBV infection/maintenance of latency. For example, IL-16 is the most abundantly expressed cytokine in MHV68-immortalized B cells of mice and upregulated upon MHV68 infection. (MHV68 shows similar characteristics to EBV.) Thus, the observed phenotype may be a result of latency maintenance. It would also explain why genes found in immune response to infection are upregulated.

We apologize for the potential confusion regarding our eQTL analysis of the GEUVADIS data, especially as related to the EBV immortalization, and thank the reviewer for encouraging us to give more information on how our study dealt with this potential confound.

We, of course, recognize that TE expression dysregulation is a feature of many types of viral infections. Consequently, we had already included the EBV expression levels as a covariate in the eQTL-related analyses, although we only mentioned it in the method section (page 34, line 962). This was all the more important that distinct LCLs may have different EBV copy numbers dating to the immortalization event. We now explicitly refer to it in the revised text and show data demonstrating that EBV expression variation is controlled for in our eQTL analysis (new **Supplemental Fig S2B**).

Initially, since we decided to use only one LCL (GM12878) for functional validation experiments, we had not previously quantified EBV levels for these analyses. Thus, to address the reviewer’s comment, we inspected EBV transcriptional levels in our RNA-seq datasets (overexpression or peptide treatment). Importantly, our data did not reveal any significant changes in EBV expression upon overexpression/manipulation of candidate regulators (all conditions with FDR > 0.05; new **Supplemental Figure S14C, S16C**), suggesting that changes in EBV levels are not the driving force for our other biological observations.

For all datasets and to increase the transparency of our analysis, we now include panels showing the lack of covariance between EBV and other elements of interest. We also highlight these additional analyses in the revised text (Page 8, Line 214; Page 18, Line 516; Page 20, Line 593).

Taken together, I feel that Bravo et al. need to reinvestigate their results and unambiguously show that they indeed find evidence for canonical L1/transposable element expression.

This point mirrors the concerns raised by other reviewers, which, as discussed above, we believe stem from the text shortcut we used in our original manuscript, conflating “transcription” and “RNA levels”, which was mistakenly interpreted as us looking only from promoter-dependent effects on TE levels. We note that in many demonstrated instances, TE RNA leads to clear biological consequences without the need for transposition or promoter-driven transcription [Marasca et al. 2022; Pasquesi et al. 2023; Ardeljan et al. 2020; Luqman-Fatah et al. 2023]. We have amended our language throughout the revised manuscript (including a new paragraph in the introduction) to make it clear that our interest lies in TE RNA levels, rather than specific transcriptional regulation. Indeed, the enrichment of TE level-related SNVs for aging and age-related disease related SNPs that we report is consistent with the biological significance of the TE-level associated identified genetic variants.

Importantly, we also now include brand new analysis based on locus-specific quantification and stratification by genomic location (exonic/intronic/intergenic) at the subfamily level for each category (new **Supplementary** **Figure S7, S9D-F, S11C-D, S15C-D** and **Figure 5F**). We have repeated our eQTL analysis using the intergenic TE expression profiles, and we have assessed whether our treatments influence intergenic profiles by GSEA. Our new analyses strengthen and expand our previous results, showing that our top candidates can influence the RNA levels of (likely promoter-dependent) distal intergenic TEs, and not just exonic/intronic proximal TEs.

We hope that this additional approach alleviates the reviewer’s concerns.

Also, within the text and figures, it would be helpful if the authors would provide more details of their results (e.g., which subfamilies were identified).

We thank the reviewer for their suggestion, and we have updated both the manuscript and the figures to highlight the specific subfamilies identified in the eQTL analyses.


**Reviewer #3 (Significance (Required)):**

The overall significance of the role of L1s, their deregulation, and possible intersection with aging is high. However, the authors do not provide evidence for L1 expression and do not engage with L1 expression versus exonization of transposable element sequences. I will be able to better assess the significance of their findings once my concerns are addressed. The target audience would be transposable element and aging researchers. Scientists interested in pipelines for eQTL analyses may also be interested in the work. I consider myself an expert in the transposable element field. I also have extensive experience in genomics with emphasis on humans.

We thank the reviewer for their careful consideration of our work, and the scientific audiences that may be interested in our new approach. We have updated our manuscript to try to engage with both canonical L1 expression and L1 expression arising from exonization, which we hope address their concerns appropriately.
