# Supplementary material for: An eQTL-based approach reveals candidate regulators of LINE-1 RNA levels in lymphoblastoid cells
Source: PLoS Genet. 2024 Jun 7;20(6):e1011311. doi: 10.1371/journal.pgen.1011311 (PMC11189215; doi:10.1371/journal.pgen.1011311)
Supplement: Attachment — Submitted filename: eQTL_Manuscript_Rebuttal_v4.docx [file pgen.1011311.s028.docx]

## **Point-by-point description of the revisions**

**Reviewer #1:**

I am satisfied with the novel version of the manuscript and the answers provided by the Authors. I believe the changes incorporated in the manuscript respond to the majority of the raised issues and have no additional comments on this beautiful piece of work.

We are pleased that Reviewer 1 finds that our revisions addressed the issues that were previously raised, and we thank them for their very kind words!

**Reviewer #2:**

Bravo et al use statistical genetics approaches to study variation in LINE-1 RNA expression in human LCLs. As evidenced by prior reviews made available, the abstract introduces LINE-1 as "autonomous, actively mobile elements" but this analysis is mostly about intronic and no longer autonomous or mobile elements transcribed as a consequence of their location in conventional gene's ranges of transcription. The introduction has been largely amended to explain this, but the abstract still points to the rare, exciting and not-exactly in view here "hot" L1s. The abstract and introduction still seemingly acknowledge that these would be the most interesting.

If that is the case, I would suggest that the authors would use this same data and the same statistical tools to perform a complementary analysis: rather than look at SNVs genome wide, analyze whether the substantially fewer L1 MEVs (Kojima et al., Nat Genet) are associated with L1 RNA expression levels, or expression levels of the portion of L1 RNA that can best indicate bona fide L1 transcription from its own promoter (McKerrow et al., NAR).

We apologize for our over-emphasis of “hot” L1s in the abstract and the introduction. Though we were agnostic regarding whether the analyzed L1s had to be mobilization-competent, we agree that our manuscript focuses a lot on interesting features of non-autonomous L1s. We have amended the abstract to highlight that the majority of L1 copies are degenerate and no longer mobile, as well as to clarify that both mobile and immobile copies were of interest in our study because of their potential impact on host biology (Page 2, line 29-32). We have also modified the introduction to explicitly indicate our agnostic interest in any L1 differences (Page 7, line 175).

Given this agnosticism, we believe that the suggested complementary analysis would be outside the scope of this paper. Nevertheless, we enthusiastically agree that the complementary analysis is the logical next step for a follow-up project. Our data identifies hundreds of SNVs that associate with differences in L1 subfamily expression. One can reasonably hypothesize that polymorphic L1s would also associate with L1 RNA pools either directly (due to transcription of the polymorphic L1s) or indirectly (by modulating the levels of genes that regulate L1). One can further expand on this analysis and include structural variants of other types, such as Alu polymorphisms, to explore intra-genomic parasitic conflicts more generally. Indeed, Kojima et al. and other groups identified an Alu polymorphism that may influence *HSD17B12* expression levels—one of the genes we considered a candidate regulator of L1 levels (although our follow-up experiments did not see a significant effect on L1 levels in GM12878 cells). Ultimately, this complementary analysis raises a lot of interesting questions and research directions that we feel are outside of the scope of the current manuscript.

As it stands, their analyses are built around an initial set of so called L1 trans-eQTLs. These would be more appropriately termed LINE1 expression QTLs, since these variants may be regulating LINE-1 RNA levels in cis- or in trans-.

We use the term “trans-eQTL” since we performed a genetic association analysis for L1 RNA levels as a group without distinguishing specific elements (*i.e*. not working on single insertion events), which will summarize effects on L1 elements throughout the genome and not just in *cis*. This is consistent with the accepted definition of trans-eQTLs [PMID: 29891877]. We have now clarified this point in the manuscript now (Page 8, line 204).

The point is that they looked genome wide for variants associated with LINE-1-mapping RNA levels. They found 3 true loci (plus two seemingly single-variant suggestive hits), and these are each interesting - something meaningful is going on with these loci and L1, but it is not clear to me what. They should analyze each locus in more granular structural genetic detail before adding on additional statistical genetics, e.g. mediation.

We now include an analysis of L1 copy number near our top loci (Page 11, line 275; new **Supplementary** **Figure S3C**). To gain insights into whether *L1* insertions near our index SNVs may be a confounding genetic factor for our study (as raised by the reviewer), we compared the number of *L1* fragments within 5 kb on either side of each index SNV and 1000 random SNVs from the study (new **Supplementary** **Figure S3C**). Importantly, the number of L1 insertions near our index SNVs was not significantly different than expected from random sampling in the genome, suggesting that index SNVs are not preferentially found in regions with substantial enrichment for L1 insertions. Moreover, for the *IL16/STARD5* index SNV, no L1P4a copy was within the 10 kb window. These results suggest that differences in L1 expression are unlikely due to differences in proximal L1 copy number.

***Supplementary*** ***Figure S3C:*** *The number of L1 fragments near 1000 random SNVs or the 5 trans-eQTL index SNVs were calculated. A two-sample Wilcoxon test was run to determine whether there were significant differences between the two groups.*

They should dedicate more time to convincing readers that these 3 loci actually impact the levels of the L1 families they say they do. To me, this would require that they include PEER factors in the "trans eQTL" analyses. If they tried, and the significance evaporated, that could be informative about the nature of these loci.

We thank the reviewer for their suggestion. When first designing this approach, we thought heavily about whether inclusion of PEER factors would be appropriate for this analysis. We opted not to include PEER factors due to a combination of analytical and technical limitations:

(1) Firstly, and most importantly, Lappalainen et al. in the initial GEUVADIS publication noted that synthetic covariates (such as from PEER) can correct out genome-wide biological effects, which can be problematic for trans-eQTL analysis where the focus is aggregate, genome-wide effects. Consequently, we included only known covariates in our eQTL analysis to avoid potentially erasing the signal we were interested in studying.

(2) Secondly, this issue of erasing real biological genome-wide effects is exacerbated by the fact that the number of PEER factors that can be included in an analytical model is arbitrary and can vary from user to user.

(3) Finally, to our knowledge, the PEER package has not been actively maintained and cannot be installed on modern versions of R, which can raise reproducibility issues if users have to setup unique environments to run PEER. (For the purpose of addressing the reviewer’s concerns, a docker image with an older version of R and the PEER package had to be used). For these reasons, we opted not include PEER factors in our final analysis.

However, since the reviewer’s worry that PEER factors may capture covariates that may confound genotype ~ gene expression relationships (an important caveat for eQTL analysis), we now also include this analysis in the revised manuscript as requested by the reviewer (new **Supplementary Figure S2C,** new **Supplementary Table S1D**). To further determine whether our identified trans-eQTL relationships are real, we re-ran our trans-eQTL analysis after regressing out 10 PEER covariates from the gene expression matrix, in addition to the previously listed known covariates (*i.e.* laboratory, population category, first 2 principal components from the SNV calls, biological sex, net Alu and L1 copy number from the structural variant data, and EBV expression levels). Importantly, after doing so, although there were differences in the absolute calculated p-values, we replicated the *trans*-eQTL associations for the top 3 loci without a substantial change in significance levels. Moreover, inclusion of the PEER factors actually led to the identification of additional loci that were not captured in our original trans-eQTL analysis. Since they may represent loci that the community may want to follow up on, we now report this analysis in the revised manuscript (Page 10, line 248; new **Supplementary Figure S2C,** new **Supplementary Table S1D**). Ultimately, our new results suggest that the top identified SNV-L1 associations are unlikely to be the mere result of unknown hidden covariates as raised by the reviewer. We thank the reviewer for suggesting this analysis, which we believe has further strengthened our findings.

***Supplementary Figure S2C:*** *A Manhattan plot for the L1 subfamily trans-eQTL analysis in the European cohort, after correcting for known covariates as well as 10 PEER factors. The genes and L1 subfamilies identified in the original analysis without PEER are listed next to the equivalent peaks in this analysis. The solid line at p = 2.44E-7 corresponds to a Benjamini-Hochberg FDR < 0.05, which was used to define significant trans-eQTLs. FDR: False Discovery Rate.*

Next, they checked which cis-eQTLs "overlap" with these 499 variants. The fact they they turn up >800,000 eQTLs suggests that they looked simply at overlapping genomic ranges.

We apologize for the confusion, as it seems that our approach was not explained clearly enough in the previous version of the manuscript. The cis-eQTL analysis for gene expression was performed on all expressed genes against all variants, not just L1 eQTL variants (see **Supplementary** **Figure S2A**). Though Lappalainen et al. did this in the original GEUVADIS publication, we re-carried out the analysis using our pipeline (i.e. including the same genome version, gene annotations, software versions, etc.) and genome-wide SNV calls/annotations that were not initially available. We chose this approach in order to have a homogenous and up-to-date set of cis- and trans-eQTLs annotations that we could easily and reliably compare. We have now updated the text to clarify this (Page 8, line 212). More importantly, we note that the overlap to L1 trans-eQTLs was only done afterwards on the final analysis file.

We have now clarified this in the revised text and methods (Page 8, line 211; Page 9, line 230).

They should instead have performed colocalization analyses. In the end, however, they land on index variants in LD blocks associated with both a certain L1 family expression and a certain gene's expression. The trivial explanation for such loci, as suggested by another reviewer, is that an L1 element of that family is present in the introns of the eGene, and for their strongest locus, this appears to be one possible (and to me, exceedingly plausible) explanation. The regression analysis is not a convincing addition.

We respectfully disagree with the reviewer there. The mediation analysis suggests that part of the effect of the SNVs on *L1* expression are mediated by *IL16/STARD5* expression. Moreover, our experimental data regarding our top candidate *IL16* clearly indicates that substantial effects on L1 levels are unrelated to the L1 intronic element as: (i) the *IL16* overexpression plasmid does not contain that intron, (ii) effects are seen with peptide treatment (again, independent of any intronic element), and (iii) we also see significant effects on distal, non-intronic *L1* levels (**Supplementary** **Figure S15C-D, Figure 5F**).

Thus, we believe the trivial explanation to no longer be plausible in the light of our follow-up validation experiments after the genome-wide eQTL analyses. We now specifically mention the intronic L1 and discuss this possibility in the revised manuscript (Page 23, line 667).

The experimental work is predicated on the statistical genetic analysis, which is not yet convincing. It should be well noted that LCL gene expression is impacted substantially by B cell activation state and EBV gene expression latency program (e.g. SoRelle et al., eLife). These could likely be captured as PEER factors, but can be missed if EBV expression is considered only as the sum of all EBV gene expression.

We agree that B cell activation state and EBV gene expression latency may influence eQTL relationships. We have attempted to address this by re-running our eQTL analysis with an additional 10 PEER covariates regressed out from the gene expression profiles (see above), as this should capture such differences. As highlighted above, despite regressing out the 10 PEER factors which may capture any residual impact of EBV activation not captured in the expression levels used as a known covariate, our analysis still captures the top 3 loci in our original analysis with no substantial changes in significance, further reasserting that the identified trans-eQTLs associations are likely to be real. To note, since our follow-up validation experiments are all performed in the GM12878 LCL line (which has a set latency/expression), and we are able to capture significant impact of the *IL16/STARD5* candidate genes, EBV activation levels are unlikely to drive the effect of our top locus. However, to address the reviewer’s concerns and since we cannot 100% guarantee that PEER covariates did capture activation state and EBV latency (since these factors were not measured at the time of sample collection, to our knowledge), we have expanded our discussion section to specify that this may be a caveat (Page 27, line 780).

**Reviewer #3:**

It is our opinion that the authors have adequately addressed reviewer concerns. In particular, the main objection raised by the reviewers having to do with a conflation between TE-promoter driven versus non-TE-promoter driven transcription was thoroughly dealt with, as is reflected in a lengthy discussion of the matter and a substantially bolstered analysis considering the genic context of TE-read origin. The revised work is much stronger for this. On the concern of whether GSEA applied to TE subfamilies is appropriate, we agree with the authors that although not commonly practiced, this is in fact the ideal tool for their stated purpose. We ask only that the authors double check whether the direction of change attributed to gene sets in figure 3 panels E-G are correct (perhaps genes were accidentally arranged in ascending Wald-statistic order?), as some of the cited literature (Wahl et al. 2020) identifies the opposite trend, namely a positive relationship between TE and mTORC1 expression. The present results are nevertheless consistent with Marasca et al. 2022 which also observe a negative relationship, but double checking should be easy enough.

Overall, we appreciate the author’s thoughtful response to the reviewer critiques. We think it makes an important contribution to the existing body of literature on the regulation of TE expression.

We are pleased that Reviewer 3 finds that our revisions have addressed their concerns and strengthened the overall analysis. We have re-reviewed the code for the GSEA analysis and find that genes were correctly arranged in decreasing Wald-statistic order. Thus, we believe Figure 3 Panels E-G accurately reflect our findings. We do agree, however, that there are what seem like inconsistencies in the literature, such as between Wahl et al. and Marasca et al. However, it is our view that, consistent with the relatively low number of consistent and shared regulators identified in the L1 CRISPR screen by Liu et al. using two cancer cell lines, these differences may reflect cell-specific TE expression responses. Indeed, Marasca et al. focused on T-cells and identified what is likely a T-cell specific regulatory program, whereas Wahl et al. studied the liver. Ultimately, we believe that more studies are needed to define cell type- and tissue-type specific TE responses across experimental treatments. We now specifically discuss our finding on mTOR pathway regulation in light of the 2 manuscripts mentioned by the reviewer (Page 16, line 456).
